# Extraction of Bioactive Compounds from Wine Lees: A Systematic and Bibliometric Review

**DOI:** 10.3390/foods13132060

**Published:** 2024-06-28

**Authors:** Filipe de Oliveira Melo, Vanessa Cosme Ferreira, Gerardo Fernandez Barbero, Ceferino Carrera, Ederlan de Souza Ferreira, Marcelo Andrés Umsza-Guez

**Affiliations:** 1 Food Science Postgraduate Program, Faculty of Pharmacy, Federal University of Bahia, Salvador 40170-100, Brazil; filipeomelo@outlook.com (F.d.O.M.); ederlan.ferreira@ufba.br (E.d.S.F.); 2 School of Food Engineering (FEA), University of Campinas (UNICAMP), Campinas 13083-862, Brazil; vanessa151ferreira@hotmail.com; 3 Department of Analytical Chemistry, Faculty of Sciences, Agrifood Campus of International Excellence (ceiA3), Wine and Agrifood Research Institute (IVAGRO), University of Cadiz, 11510 Puerto Real, Spain; gerardo.fernandez@uca.es (G.F.B.); ceferino.carrera@uca.es (C.C.)

**Keywords:** phenolic compounds, ultrasound, waste, wine, anthocyanins

## Abstract

The extraction of bioactive compounds from wine lees involves a variety of methods, the selection of which is crucial to ensure optimal yields. This systematic review, following PRISMA guidelines and utilizing the Web of Science database, aimed to examine the current state of this field, providing insights for future investigations. The search employed strategies with truncation techniques and Boolean operators, followed by a three-step screening using well-defined eligibility criteria. A bibliometric analysis was conducted to identify authors, affiliations, countries/regions, and research trends. Thirty references were selected for analysis, with Spain standing out as the main source of research on the topic. The majority of studies (66%) focused on the extraction of bioactive compounds from alcoholic fermentation lees, while 33% were directed towards malolactic fermentation lees. Binary mixtures (ethanol–water) were the predominant solvents, with ultrasound being the most used extraction method (31.3%), providing the highest average yields (288.6%) for the various evaluated compounds, especially flavonoids. The potential of wine lees as a source of bioactive compounds is highlighted, along with the need for further research exploring alternative extraction technologies and the combination of methods. Additionally, the importance of “in vitro” and “in vivo” tests to assess the bioactive potential of lees, as well as the use of computational tools to optimize extraction and identify the molecules responsible for bioactive activity, is emphasized.

## 1. Introduction

By-products from the wine industry represent a percentage ranging from 31 to 40% of the total harvested grapes [1]. Among these, fermentation residues (lees) represent on average 25% of the waste generated during the winemaking process and, if utilized, could contribute to improving the economic and environmental sustainability of winemaking activities, mainly due to their application as ingredients in enriched food and cosmetic formulations [1,2,3,4].

Wine lees have an average chemical oxygen demand (COD) of 30,000 mg/L [5] and can be classified, depending on the stage of the process in which they are formed, into first (alcoholic) and second (malolactic) fermentation lees, as well as aging lees, when formed during the maturation process in wooden barrels [6]. Jara-Palacios [7], in turn, also classifies lees according to the size of the particles formed, i.e., as heavy, when they have sizes ranging from 2 mm to 100 μm, and light lees, with sizes smaller than 100 μm. In any case, this by-product is considered an important source of bioactive compounds that, when reused, become a relevant economic and alternative source for the wine segment.

Regarding its composition, wine lees have an organic matter content ranging from 598 to 936 g/Kg and high concentrations of oxidizable organic carbon, between 226 and 376 g/Kg [5]. Additionally, studies have identified high concentrations of polyphenols in its composition [7]. These compounds, due to their phytotoxic nature, can pose a significant environmental threat if not properly treated [8], potentially causing various impacts, such as the disruption of plant photosynthesis and transpiration cycles and a reduction in soil fertility [9].

Despite this, the consumption of these phytochemicals is associated with a range of beneficial bioactive effects in the body, including the regulation of lipid metabolism [10], modulation of the gut microbiota [11], and control of hypertension [12]. These benefits, for example, have led to the application of wine lees in a variety of products, as demonstrated in a prospective study by Bulos et al. [3] and in research exploring their use in bread [13], biscuits [14], ice cream [15], and cereal bars [16].

In addition to the above, it is important to highlight the variety of phenolic compounds present in wine lees, as different subclasses have already been identified, such as phenolic acids, flavonols, and flavonoids, with anthocyanins being one of the main representatives [4,7]. These compounds can even be extracted and isolated from this residue using different extraction techniques.

In this context, the technologies involved in extraction processes comprise a set of mechanisms and procedures to release the target compounds from the matrix in which they are contained. These procedures are influenced by a wide variety of factors, ranging from the characteristics of the raw material and the nature of the target compound to the properties of the solvent used [17]. These factors have an impact on the choice of the appropriate extraction method, which can be categorized as conventional, innovative, or combined [18].

The selection of these methods is essential in the process of extracting these target compounds, and, in this scenario, systematic review studies emerge as a crucial tool [19]. These studies provide an overview of the state of the art, enabling the identification of new priorities, as well as problems that can be addressed in future research [20].

Thus, the objective of this systematic review is to evaluate the current state of the art regarding the main countries and researchers investigating the extraction processes of bioactive compounds from wine lees, as well as to identify possible opportunities and insights that enable the development of new research.

## 2. Results and Discussion

### 2.1. Study Selection and Flowchart

**Figure 1:**
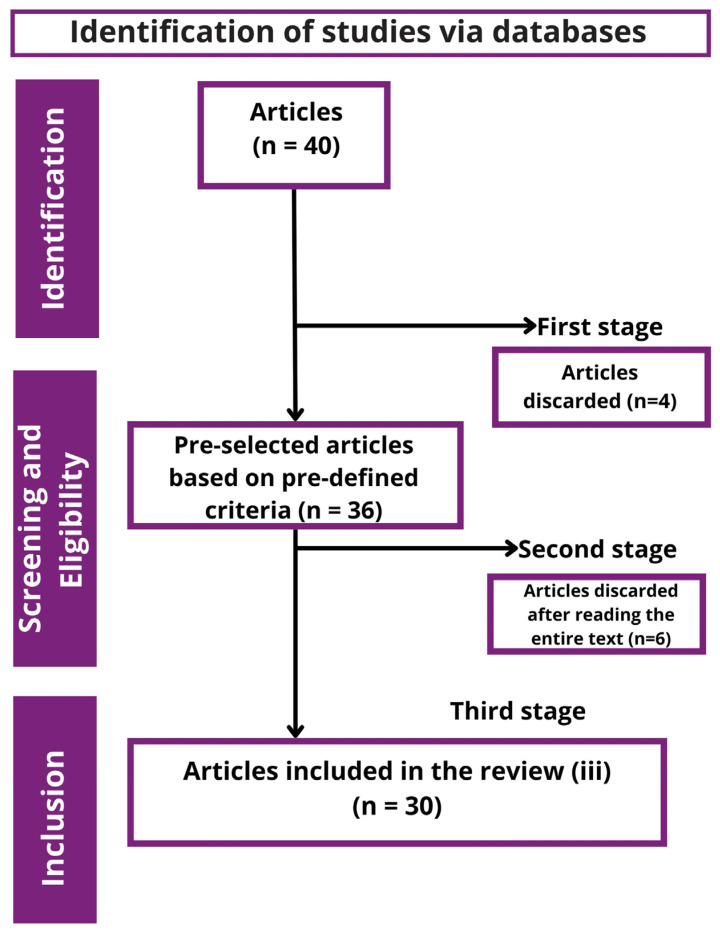
Flowchart of the article selection process.

The results of the selection are shown in Figure 1. After searching the database (Web of Science), a total of 40 scientific articles with the keywords mentioned in Section 2.2 were found.

After applying all exclusion criteria in specific stages and reading all articles in full, a total of 30 references were selected as valid texts for the development of this research.

### 2.2. Bibliometric Review

**Figure 2:**
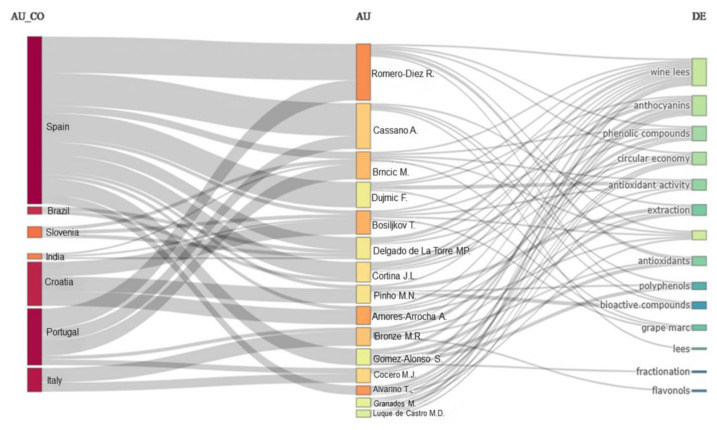
Regions, researchers, and recurring terms in research related to methods of extracting bioactive compounds from wine lees.

The selected files underwent bibliometric analysis. The chosen studies are predominantly linked to seven main countries (Figure 2), with Spain leading research related to extraction using wine as a raw material, followed by Portugal, Croatia, and Italy. This result was expected due to Spain’s importance in the global wine industry, being the third-largest wine producer in the world, with a production of around 30.7 million hectoliters in 2023 [21]. Additionally, it is important to highlight that nineteen of the selected studies were conducted at renowned research centers, such as the University of Córdoba, University of Cádiz, and University of Barcelona, which may have influenced these results.

The collaborative relationship between countries can also be observed in Figure 2, where a trend of partnership development among researchers is identified. An example is the work of Romero-Diez, which reveals partnerships between Spain and Portugal, while Dujmic established collaborations in Spain, Croatia, and Slovenia. The most recurring terms in the evaluated works include “wine lees”, anthocyanins, antioxidants, and circular economy.

**Figure 3:**
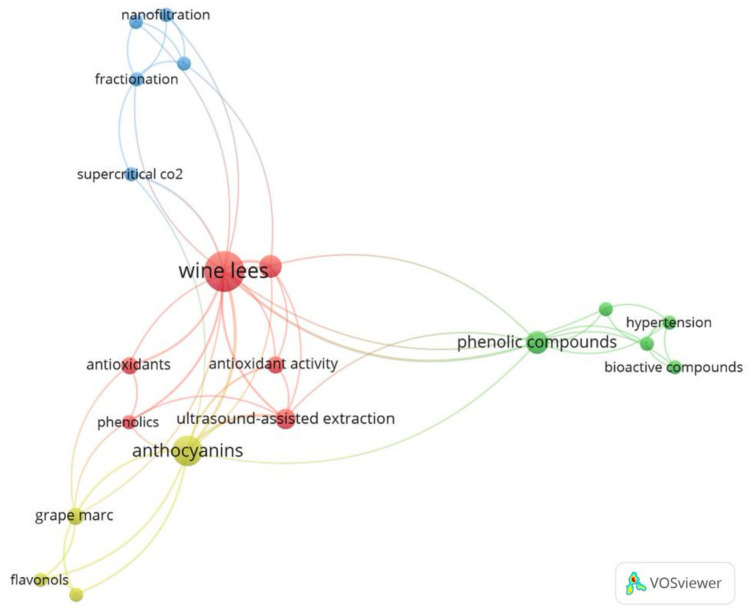
Profile of recurring keywords in research related to methods of extracting bioactives from wine lees.

Figure 3 displays the main keywords used by the authors. Terms are represented by spheres, and the larger the circumference, the higher the frequency of occurrence in the analyzed works. Additionally, terms exhibit interactions with each other; the more connections, the greater the interconnection with other terms. Different colors indicate clusters, formed by terms related to a specific area of study. As observed, the term “wine lees” is centralized, standing out with the largest sphere and the highest number of connections. These results were expected, considering that “wine lees” are the predominant raw material in the works. In the conducted research, four clusters were identified: the red one, related to biological activities; the yellow one, associated with bioactive compounds, such as anthocyanins and flavonoids; the green one, focused on the identification and application of compounds; and, finally, the blue cluster, referring to extraction methods, which includes certain terms, like “nanofiltration” and “supercritical CO_2_”.

**Figure 4:**
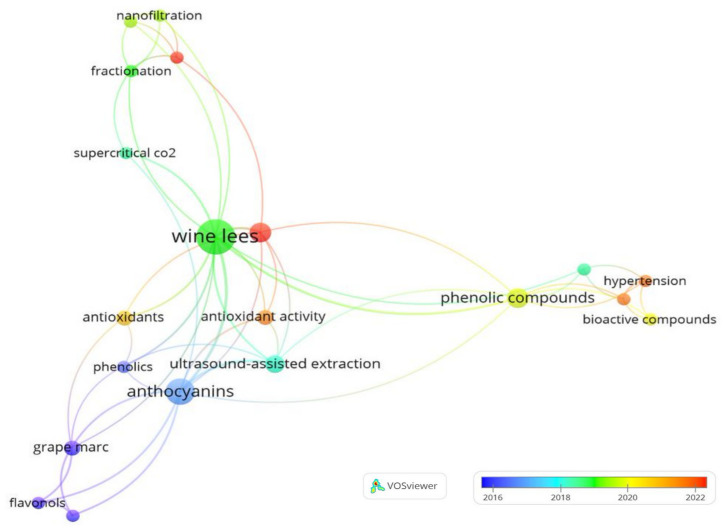
Variation in keywords applied in research on the extraction of bioactive compounds from wine lees over the years.

Figure 4 displays the keywords according to their variation over the years. Initially, research related to “wine lees” was concentrated on the extraction of bioactive compounds. However, over the years, an evolution was observed, evidenced by the need to explore new extraction methods and applications. This transition is noticeable when analyzing the terms used over time. Initially, terms associated with specific bioactive compounds were more frequent; however, as time progressed, there was a shift towards exploring new extraction methods and their applications.

**Figure 5:**
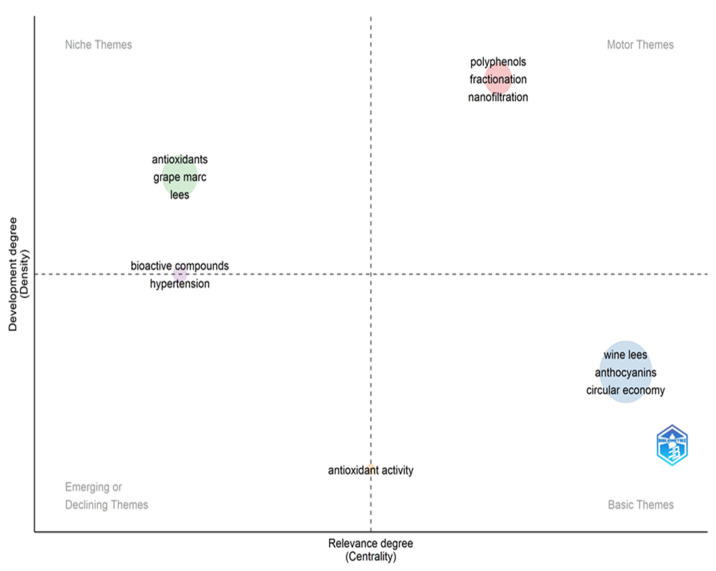
Thematic mapping of the main research topics involving the extraction of bioactive compounds from wine lees.

Figure 5 illustrates the relationship between the centrality and density of the most frequent terms in the analyzed works. Terms are grouped into spheres, where the larger the sphere, the more frequently the grouping was used. The diagram presents four distinct areas. Terms classified as “motor themes” are robust and of great importance, highlighting, for example, polyphenols. In the group of “niche themes”, terms with high density and low centrality are found, indicating that these terms are specific and fundamental to the research area, such as bioactive compounds. In the last area, “emerging or declining themes” are presented, representing topics that have emerged and have been recently addressed, but which are not very present in the literature, such as hypertension. Lastly, “basic themes” represent highly relevant terms, such as “wine lees”, “anthocyanins”, and “circular economy”.

### 2.3. Systematic Review

The specific data regarding the studies identified in this research are available in Table 1. Initially, it is noted that, concerning the types of lees used in the research, approximately 66% of the studies (that mentioned the stage of lees collection) were conducted with wine lees from alcoholic fermentation. The other 33% of the works extracted bioactive compounds from lees resulting from malolactic fermentation, also known as lees from the second fermentation.

**Figure 6:**
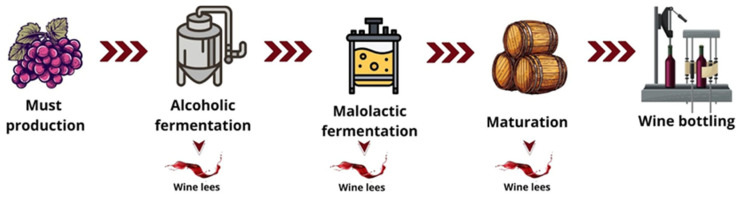
Main unit operations associated with the production of lees in wine.

Figure 6 presents the main unit operations responsible for the separation of wine lees generated during processing. As previously described, the stage at which these lees are generated represents a crucial and relevant parameter in the quest for greater extractions of bioactives from this byproduct. This is mainly because lees resulting from alcoholic and malolactic fermentations have distinct levels of phenolic compounds [51]. In an analysis of this context, studies conducted by Mir-Cerdà et al. [47] and Umsza-Guez et al. [36] pointed to malolactic fermentation lees as an important source of phenolic acids and anthocyanins, with up to twice as many phenolic compounds as lees obtained in alcoholic fermentation [51]. However, this byproduct was only used in 33.3% of the studies found in this review, indicating an important niche for future research.

It is important to mention that the wine lees used in the various works identified in this research were subjected to different pretreatments, with emphasis on prior drying of the samples. In 50% of the studies reviewed, samples were freeze-dried before the process, which resulted in significant increases in the extraction yields of bioactive compounds compared to conventional convection drying methods [32,36,50]. On the other hand, convective methods, used in 23.33% of studies, led to lower extraction rates of bioactive compounds, as reported by Umsza-Guez et al. [36], possibly due to the applied temperatures varying between 30 °C and 50 °C.

In Table 1, it is also possible to initially identify that the solvents most commonly used for the extraction of phenolic compounds from wine lees consist of a binary mixture of ethanol and water (70% of articles), in different proportions, including for different techniques. Such a result is based on the fact that binary mixtures, based on water and environmentally friendly and non-toxic organic solvents, tend to ensure high performance in the extraction process of polyphenols from different matrices [52], and this is favored by the different properties that each of the solvents possesses, mainly regarding its polarity. An example of this is ethanol, which increases the polarity of the medium [33] and consequently the levels of interaction with different molecules.

It is important to highlight that ethanol and water are solvents that can be used in the formulation of a variety of products, including food, due to not causing harmful effects to organisms when incorporated, and they are even considered as “green” solvents [53]. In this context, hydroalcoholic extracts from grape residues have shown a wide range of bioactivity, including antioxidant and antimicrobial qualities [53,54], favoring their application in products, such as toothpaste [55], chicken pâtés [56], and food packaging [57], among others.

Such applications are limited when using methanol as an extraction solvent, for example. This solvent has a considerable toxic effect [58]; despite this, it was still applied in 10.8% of the experiments that used solvents, either alone or combined with other substances.

In the context of searching for alternatives to certain solvents, like methanol, and considering the increasing environmental concerns, deep eutectic solvents [59,60] have gained ground. Such solvents are classified as liquid salts and have certain properties, such as low vapor pressures and low flammability, as well as the possibility of being designed for specific purposes [61,62]. In this research, studies that applied deep eutectic solvents, more specifically choline chloride acidified with malic acid for the extraction of bioactive from wine lees were also found, but these represented only 3.8% of the applied research. This demonstrates that despite gaining ground in recent years, the solvent’s application in residues from the wine industry is still in its early stages.

Finally, another extraction solvent identified in this study and characterized as sustainable was supercritical CO_2_. This solvent accounted for a proportion of 3.8% of the solvents used in the extraction of bioactive compounds from lees. Further details on these solvents, along with detailed information on the technique employed, will be addressed in the section dedicated to discussing the extraction process with supercritical fluid.

### 2.4. Methods of Extracting Bioactive from Wine Lees

**Figure 7:**
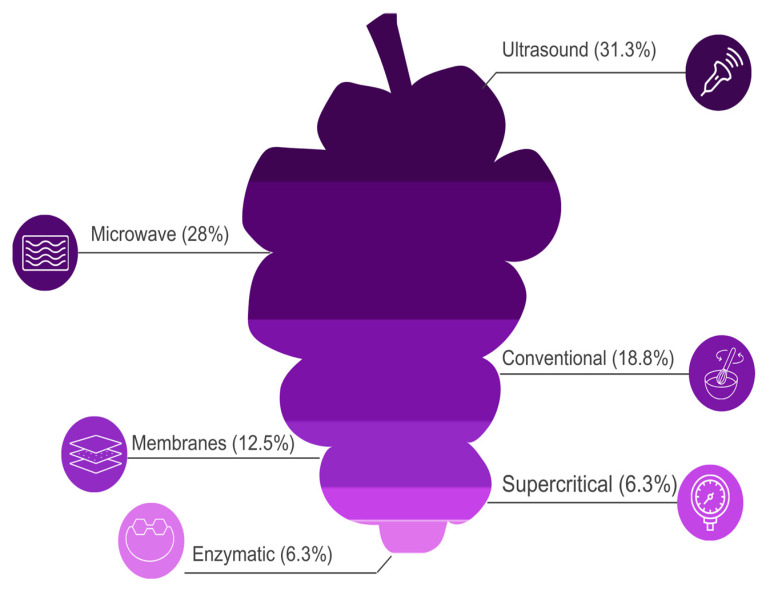
Extraction methods applied to wine lees found in the systematic review.

In general, Figure 7 presents the main extraction methods found in this research, and as can be observed, the use of ultrasound received prominence compared to other technologies, such as microwaves, conventional methods (maceration), the use of membranes, and enzymatic and pressurized supercritical fluid technologies.

The next steps of this review will be subdivided into three parts. Initially, the conventional method of extracting bioactives from wine lees will be addressed. Next, the emerging methods identified throughout this review will be presented. Finally, insights into new research being conducted in this field will be provided.

#### 2.4.1. Conventional Extraction (Maceration)

Extraction procedures encompass a variety of approaches, each designated for specific purposes, aiming at the extraction of biologically active compounds from plant material. Such methods are based on the extraction effectiveness provided by the solvents employed, as well as the heating and/or agitation techniques used [18].

Conventional extraction techniques are diverse (maceration, hydro distillation, infusion, digestion, etc.), and each has advantages and limitations depending on sample destruction, the loss of volatile components, and large solvent quantities, among other factors that can directly impact their efficiency in extracting bioactive compounds from different matrices [63,64].

These conventional methods, despite being simpler to apply and execute, and which often do not require specialized equipment and personnel, have several disadvantages, including excessive process time and the large quantity of solvents used [65]. Furthermore, low extraction yields of target compounds can also be an important limitation of these methods [18].

In the case of wine lees, the main conventional technique observed, after a full-text reading process of the articles, was precisely the maceration technique with different solvents, accounting for 18.2% (*n* = 6) of the identified studies. In this technique, the material is ground to increase the surface area and facilitate mixing with the solvent used (such as ethanol or methanol, for example). Additionally, filtration, pressing, and agitation steps occur to increase diffusion and favor the extraction of target compounds [66].

Most of the research found here presented various objectives, ranging from the characterization of different residues from the wine industry [23,24] to the extraction and encapsulation process of anthocyanins from wine lees [25].

In this context, Costa et al. [27] and Zhijing et al. [22] used an ethanol–water mixture (50:50, *v*/*v*) to extract biologically active compounds from red and white wine lees. In both studies, this solvent solution demonstrated efficacy in removing a variety of compounds, including total phenolic compounds and total flavonoids. Costa et al. [25] focused on red (Touriga Nacional, Touriga Franca, Tinta Roriz) and white (Rabigato, Malvasia Fina, Viosinho) wine lees, while Zhijing et al. [22] characterized lees from Pinot Noir (red), rosé, and white grapes. The concentration of total phenolic compounds varied among different lees, highlighting that the processing technology and the contact time between wine and grape solids influence the transfer of phenolics to the lees (red > rosé > white) [22].

In another study [26], an acidified ethanol–water solution (75:25, *v*/*v*) was used to remove phenolic compounds from Malbec wine lees. Although not quantified, the technique (solvent mixture with lees for 60 min at 50 °C) managed to extract 57 different types of phenolic compounds, including flavonols and pyranoanthocyanins, pigments derived from anthocyanins and formed through the interaction of anthocyanins with yeast intermediate metabolites (pyruvic acid and acetaldehyde) [67]. These results indicate that wine lees are a valuable source of bioactive ingredients, both from grapes and when generated during vinification.

It is important to note that some of these studies also determined the antioxidant activity of the extracts by different in vitro methods (DPPH, ABTS, FRAP, ORAC) and identified distinct potentials depending on the characteristics but directly related to the content and types of phenolic compounds present in the samples [22]. Nevertheless, the preparation of extracts using a binary mixture (ethanol–water) in concentrations higher than 50% shows promise for producing phenolic-rich extracts with high antioxidant activity. However, it is crucial to emphasize the need for further studies, including the development of new experimental designs, as developed by Tagkouli et al. [37] and Umsza-Guez et al. [36], to enhance the extraction of bioactives from various lees. This should be performed while considering the various processing variables, such as temperature, solid–solvent ratio, and solvent concentration, among others.

Despite the importance of conventional extraction methods (maceration), the use of emerging technologies, aiming to optimize the extraction of bioactive compounds from wine lees, has been gaining prominence over the years. And, as shown in Figure 6, alternative methods accounted for a total of 84.4% of the research. Specifically, in the evaluated period, mainly studies applying ultrasound (*n* = 10, 31.3%), microwave use (*n* = 9, 28.1%), enzyme use (*n* = 2, 6.3%), membrane use (*n* = 3, 31.1%), or combined methods (*n* = 2, 6.3%) for extracting bioactive from different wine lees were identified. These methods will be presented and discussed in the following topics.

The next sections will discuss the main methods found to extract bioactive compounds from lees, focusing mainly on their limitations and potentialities to support researchers in new investigations.

#### 2.4.2. Ultrasound-Assisted Extraction

Ultrasound-assisted extraction (UAE) has gained prominence as a “green” extraction method, mainly because it is often used as a pretreatment in the extraction of polyphenols from various plant matrices. This is due to the numerous advantages it presents compared to conventional methods, such as high yields, reduced or zero solvent consumption, operational ease, and lower investments [33,68,69]. Furthermore, increased extraction yield and reduced operating temperature are factors that contribute to this being a cheap, simple, and efficient technique [70].

Despite this, this technique also has limitations, such as the dependence on the nature of the food matrix regarding the effect of ultrasound and the fact that the effects of the waves are often limited to the proximity of the ultrasonic emitter [70]. These factors need to be taken into consideration when applying the technique.

This approach is based on the transfer of heat and mass induced by ultrasonic waves, triggering the ultrasonic cavitation effect, whose intensity is influenced by various factors. These include the power employed, the type and quantity of solvent used, the amplitude and geometry of the probe, the time and temperature of application, as well as specific material characteristics, such as dry matter content and sample size [33,71]. All these elements can directly or indirectly impact the extraction process yield depending on how they are employed [72].

The scientific community has been dedicated to studies optimizing ultrasound-assisted extraction (UAE) processes of compounds of interest found in wine lees. This was evident in this research, as between 2013 and 2023, 33.3% (*n* = 11) of the studies developed and indexed in the Web of Science applied this technique (alone or combined) to different wine lees.

In this context, Barcia et al. [32] evaluated the occurrence of low-weight phenolics in wine lees extracts (Cabernet Sauvignon and Cabernet Franc) obtained through sonication with a mixture of methanol, water, and formic acid (50:48.5:1.5 *v*/*v*), identifying a total of 19 anthocyanins, 9 pyranoanthocyanins (6 vitisin type and 3 hydroxyphenyl type), and 18 flavonols (12 glycosides and 6 aglycones) in their composition. Additionally, the presence of trans-resveratrol was verified in lees obtained from Cabernet Sauvignon grapes, compounds with important bioactive properties [73].

Similarly, by employing ultrasound on lees from red wine (Merlot and Vranac), Dujmić et al. [29] observed a remarkable increase in the extraction yield of various compounds, including phenolics, such as trans-resveratrol glycosides (269.3%), trans-resveratrol (9.39%), quercetin (3.89%), and kaempferol (7.6%), when compared with conventional extraction methods. The researchers also found that the use of ultrasonic probes (diameters of 22 mm, amplitude of 90%, for 1361 s (Merlot) and 1500 s (Vranac)) resulted in increased extraction of several anthocyanins present in lees, such as petunidin-3-glucoside (23.42%), malvidin-3-glucoside (41.54%), malvidin-3-(6-O-acetyl)glucoside (25.9%), and malvidin-3-(6-O-p-coumaroyl)glucoside (8.16%).

Bosiljkov et al. [28], aiming to optimize the extraction of anthocyanins from Merlot wine lees using eutectic solvents (choline chloride acidified with malic acid), identified that the use of higher ultrasonic powers (341.5 W) for 30 min was able to increase the extraction yield of anthocyanins by about 54.84%. Furthermore, Tao et al. [33], applying ultrasound frequencies in the order of 40 kHz to hydroalcoholic extracts (43.9 to 51.5%) of wine lees (Cabernet Sauvignon 60%, Merlot 30%, Cabernet Franc 10%), obtained an increase in the yield of phenolic compounds and anthocyanins by 16.5% and 20.5%, respectively, with the application time varying between 25 and 36 min, respectively. The authors attribute this increase to the cavitation phenomenon generated by ultrasound that promotes the diffusion of internal phenolic compounds into the solvent, increasing their concentration at the end of the extraction process.

The positive effects of ultrasound were also observed in the study developed by Romero-Díez et al. [39], who, despite not identifying an increase in the extraction yield of target compounds when the technique was applied, observed a 66% reduction in the extraction time of these compounds (from 15 to 5 min). In another study [31], by applying sonication for 10 min at 70 kHz to dehydrated wine lees, extracts with high levels of total phenolic compounds (254 ± 24 mg gallic acid equivalent (GAE)/g) and flavonoids (16 ± 1 to 146 ± 5 mg equivalent to catechin (ECAT)/g), correlated with high antioxidant activity measured by different methods, were obtained.

Extracts rich in phenolic compounds were also observed by De Luca et al. [34] by pretreating the lees with ultrasound. Among the results obtained, the authors highlight the possibility of producing extracts with higher concentrations of phenolic compounds (4.61 ± 0.09 to 49.56 ± 0.56 mg GAE/g) and total flavonoids (1.54 ± 0.07 to 20.15 ± 0.32 mg ECAT/g) in lees obtained from the fermentation of Magliocco Canino grapes and treated with ultrasound.

It is important to highlight that the applications of the developed extracts, including with the assistance of ultrasound, are diverse. In this context, Duarte et al. [30] explored the application of ultrasound in extracting bioactive compounds from red wine lees to assess their cosmetological potential. Using a mixture of ethanol–water (80:20, *v*/*v*), the study developed extracts with average phenolic compound contents of 37.54 mg GAE/g extract. The extracts exhibited variable antioxidant capacity and demonstrated bacteriostatic and fungistatic activity against various strains. Notably, in tests with skin cells (HaCaT and HFF-1 fibroblasts), the extracts showed a reduced cytotoxic effect, with higher cell viability at concentrations of up to 50 μg/mL. However, despite these promising findings, further studies are needed to evaluate the effects at higher concentrations on cellular cytotoxicity.

**Figure 8:**
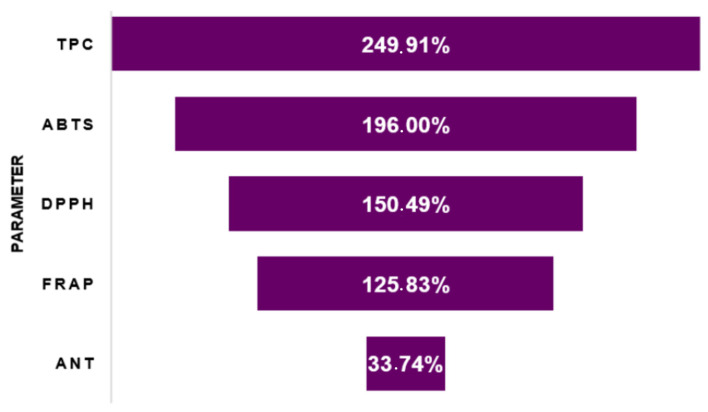
Average yield of the main compounds and bioactive properties extracted from wine lees with sonication assistance.

Furthermore, it is observed that most studies that optimized the extraction of phenolic compounds from different wine lees, using ultrasound as a pretreatment, reported significant increases in the content of bioactive compounds after treatment. This behavior was observed in certain parameters, such as total phenolic compounds (TPC) and anthocyanins (ANT) (Figure 8), as the application of ultrasound resulted in increased extraction yields of these biomolecules by 249.91% [24,29,33,34] and 39.00% [28,29,33], respectively.

Additionally, there was an increase in the in vitro antioxidant activity (ABTS, FRAP, DPPH) of the developed extracts [29,34], surpassing 100%, on average, as shown in Figure 8. This behavior was expected due to the increased extraction of compounds with biological activity, resulting, for example, in an increase in antioxidant activity.

Based on the above, it has been demonstrated that the use of ultrasound has mainly contributed in two aspects: (i) reducing extraction time and (ii) the increase in the extraction yield of bioactive compounds, when compared to conventional methods for extracting bioactives from wine lees. These positive impacts are directly related to the phenomenon of ultrasonic cavitation, which, by breaking the cell wall, favors mass transfer processes, causing a greater release of polyphenols from the matrix in a shorter time [74].

However, studies in this area are still limited, especially with regard to information about the other benefits of using ultrasound on wine lees. Among these benefits, the reduction in the use of solvents, the improvement in the quality of the extracts produced, and the reduction in environmental impact compared to conventional methods stand out [75]. These gaps need to be filled through new research.

#### 2.4.3. Microwave-Assisted Extraction

Microwave-assisted extraction is a method in which microwave energy is used to heat polar solvents in contact with solid samples, facilitating the partitioning of compounds of interest between the sample and the solvent. This process results in a significant reduction in extraction time and solvent consumption [76]. Moreover, when applied to certain materials, such as vegetables and yeasts, microwaves provide, through high temperatures, the disruption of the structure of these materials, promoting greater release of a wide variety of compounds, including anthocyanins that are interacting with these structures [77].

Thanks to the efficiency of microwaves, the heating process occurs in a matter of seconds [42], such that the high temperatures reached after irradiation tend not to cause phenolics degradation due to the short duration of heating [42].

It is important to highlight that in recent years the application of this technology has been driven by certain factors, such as its ability to be applied directly to the desired biomass, without the need for solvents or pretreatment, such as drying, its ability to provide rapid and uniform heating, and its adaptability for continuous and easily scalable processes [78]. Despite this, this technology has limitations, such as low extraction yields of volatile compounds at higher temperatures, the limited number of solvents that absorb microwave energy [79], and the possibility of changes in the field dielectric of macromolecules, which could favor the breaking of hydrogen bonds with consequent rupture of their structure [80].

Despite this, in the period examined (2013 to 2023), eight studies (25.80%) were identified that used microwaves at some stage of the process in order to optimize the extraction of bioactives from wine lees of different varieties.

One of the earliest identified works was developed by Delgado de la Torre et al. [43]. The authors applied microwaves at a power of 140 W/10 min in hydroalcoholic extracts (60:40, *v*/*v*, acidified with HCl, pH 4) obtained from lees of different varieties (Tempranillo, Mazuelo, Graciano, Garnacha, Syrah, Cabernet Franc, and Merlot). The applied treatment showed high variability in the content of anthocyanins in the developed extracts (0.09 to 77.5 ng/g), correlating this behavior with certain factors, such as the type of lees used (liquid or solid), grape variety, vinification practices, and storage conditions, as well as the region where the grapes are produced.

The processing conditions applied in the previous paragraph were also used in the work of Delgado de la Torre, Priego-Capote, et al. [38] and Delgado de la Torre, Priego-Capote, et al. [40] when obtaining extracts from first fermentation wine lees (Tempranillo, Mazuelo, Garnacha, Cabernet). Similar to what was reported earlier, both studies extracted and identified a wide variety of compounds in the obtained extracts, with the presence of gallic acid, procyanidin B2, pelargonidin 3-(6-p-coumaroylglucoside), conjugates, such as malvidin 3-galactoside, as well as the presence of some flavonols, such as quercetin and myricetin, being highlighted. In addition to these compounds, Delgado de la Torre, Priego-Capote, et al. [38] also found the presence of a primary amino acid in all analyzed samples, namely valine.

Tagkouli et al. [37] optimized the extraction conditions of bioactive compounds from lees of wines of various white grape varieties (Kidonitsa, Savvatiano, Chardonnay, Moschofilero) and red (Red Grenache, Merlot, Cabernet, Agiorgitiko) from different wineries in Greece. They found better extraction conditions when lower microwave powers were applied (54 W/35 min, ethanol–water 50:50, *v*/*v*). The extracts obtained from red wine lees showed, in addition to higher levels of total phenolic compounds, ranging between 8.0 (±1.4) and 26.0 (±1.1) mg of GAE/g of dry sediment, higher antioxidant activity (FRAP and ABTS), as expected, when compared to white wine lees. The extracts also demonstrated greater antimicrobial potential, especially against B. aureus strains. In this context, it is important to highlight that the results obtained in this research were mainly influenced by the origin and technological stage where the lees was collected (pre- or post-fermentation).

Meanwhile, Matos et al. [42], when preparing hydroalcoholic extracts (60:40, ethanol–water) from wine lees (Tempranillo) (power of 300 W/1.5 min, at 115 °C), observed extraction yields of phenolic compounds exceeding 10%, when compared to conventional solid–liquid extraction without the use of microwaves. Moreover, the application of microwaves increased the antioxidant potential of the obtained extracts, as the authors observed increases of 10% (ORAC), 29% (HORAC), and 35% (HOSC) in this activity depending on the applied in vitro methodology. It was also observed that extracts from red wine lees exhibited a greater inhibitory effect on elastase, tyrosinase, and matrix metalloproteinase-1 (MMP-1), enzymes important in skin aging processes [81].

Furthermore, regarding the work developed by Matos et al. [42], it is important to note that the authors considered working under high-power conditions (300 W) for short periods of time (<120 s), with the aim of ensuring the thermal effect of microwaves, without causing the degradation of active compounds present in the samples, as reported in other studies [82,83] and evaluated by Romero-Díez et al. [39]. In the conditions studied (300 W/1.5 min), the later authors were able to obtain a maximum extraction yield of phenolic compounds in the order of 49.50% and anthocyanins greater than 100%, when compared to the conventional method (50% ethanol, for 15 min at 25 °C).

Finally, Ciliberti et al. [41] worked with microwave frequencies in the order of 2450 MHz, evaluating the effects of solvent types (ethanol and water), temperature (50, 100, 150, and 200 °C), and the presence of sodium carbonate Na_2_CO_3_ (2 mmol/g of dry weight of lees) on the increase in extraction yield of polyphenols in red (Nero di Troia), rosé (Nero di Troia), and white (Trebbiano) wine lees. The authors observed that higher temperatures (200 °C) were able to positively influence the process, increasing the extraction yield of phenolic compounds for all analyzed samples (white—increase of 190%, rosé—increase of 120%, red—increase of 57%). Moreover, the type of solvent also symbiotically impacted the extraction rates of these compounds, with the ethanol–water 50% mixture without the use of sodium carbonate Na_2_CO_3_ standing out, as its presence did not impact the extraction yield levels, except for the lees obtained from red wine.

Considering the studies conducted so far, the application of microwaves emerges as a promising technique in optimizing the extraction of bioactive compounds from various wine lees. Similar to ultrasonication, this approach also enhances the antioxidant capacity of the resulting extracts, especially at higher powers. However, it is crucial to monitor the extraction time to ensure the stability of the compounds, while benefiting from the temperature increase to improve the extraction properties of the solvents used.

Moreover, it is crucial to highlight the importance of comparing the proposed method with traditionally employed methods, evaluating its effectiveness in extracting compounds compared to established standards. The lack of this practice has been observed in many studies, which can result in biases that compromise the quality of research.

Finally, it should be noted that microwaves are also being used in conjunction with other methods to optimize the extraction of bioactive compounds from different wine lees, such as ultrafiltration. These works will be presented in the section dealing with methods of extraction and the purification of bioactive compounds from wine lees by membranes, in Section 2.4.5.

#### 2.4.4. Enzyme-Facilitated Extraction

As previously discussed, it is known that wine lees consist of a combination of dead yeast/bacteria (depending on the type of wine), their metabolites, and phenolic compounds, as well as tartaric salts and plant material (grapes) [84]. Phenolic compounds mainly originate from grapes and are absorbed by microorganisms during the biotechnological process. Most bioactive compounds are connected to different structures through hydrophobic, hydrogen, or even esterified bonds with biomolecules, such as proteins and carbohydrates, for example [85].

Thus, aiming to release these compounds from these structures, a wide variety of extraction methods have been applied [86,87], including enzymatic methods [88]. Enzymes are catalysts that hydrolyze certain compounds, such as proteins, cellulose, lignin, hemicellulose, and pectins from the cell wall of different food residues, releasing a wide variety of compounds into the medium [74,89,90,91], even allowing their separation and purification.

There are several enzymes that can be applied in enzymatic extraction processes, with an emphasis on cellulases, pectinases, and hemicellulases [89]. And, more recently, proteases have been applied with the aim of increasing the extraction of bioactive compounds from a wide variety of products [92,93]. Their applicability is centered on the weakening or disruption of cell walls and membranes, generating an increase in their permeability [74,94].

Among the advantages of these biocatalysts, the high specificity, the catalytic action in a shorter time and the mild processing conditions stand out [95]. Furthermore, they are environmentally friendly compounds that can be used in a wide variety of products without causing significant environmental impacts [74]. However, the main disadvantages include their high cost and low commercial popularity, which has hampered their global industrial application, often limiting their use to fruit juice extraction processes [95].

In the case of wine lees, this technology is still considered “new” compared to other methods, such as ultrasound and microwaves, for example. This is because, of all the methods found in this research in publications in the Web of Science in the period between 2013 and 2023, only 6.3% (*n* = 2) of the works employed applied enzymes in processing for this purpose.

One of these works was developed by López-Fernández-Sobrino et al. [44], who applied the Flavourzyme^®^ protease (EC 3.4.11.1, leucine aminopeptidase (LAPU)/g of *Aspergillus oryzae*) with the aim of generating a proteolytic hydrolysis of the cell wall present in wine lees (first fermentation, cabernet) and increasing the extraction yield of phenolic compounds, also evaluating their antihypertensive potential before and after treatment. In the study, the application of the enzyme, in the proportion of 80 LAPU/g protein at 25 °C for 2 h at pH 4.0, provided an increase of about 55% in the content of phenolic compounds in the wine lees extract. In addition, the authors observed a 22% increase in catechin content (from 2681.20 ± 19.20 to 3289.60 ± 20.80), 119% in quercetin (from 888.40 ± 4.80 to 1954.40 ± 9.20), 87% in malvidin-3-glucoside (from 1780.76 ± 20.01 to 3334.75 ± 37.47), and 121% in cyanidin-3-glucoside (from 21.72 ± 0.81 to 48.11 ± 1.79). In addition to this, there was an increase in the concentration of some phenolic acids, such as gallic acid, and of amino acid residues, such as proline, leucine, and valine, for example.

It is important to highlight that the hydrolyzed extracts obtained by López-Fernández-Sobrino et al. [44] still showed potential for controlling arterial hypertension when applied in the inhibition of important enzymes in arterial hypertension processes (ACE—angiotensin-converting enzyme), in addition to acting by inhibiting blood pressure in in vivo models (hypertensive rats). Thus, according to the authors, enzymatic treatment proves to be a good strategy for releasing phenolic compounds from lees, even enhancing their antihypertensive effect.

In another work [45], using the same enzymatic extract mentioned above, protein hydrolysates rich in peptides with bioactive and antihypertensive properties were obtained from wine lees. After an isolation process, six of the peptides were identified, and three of them showed angiotensin-converting enzyme (ACE) inhibitory activity (IC 50) lower than 20 µM, in addition to in vivo studies that demonstrated the potential of wine lees (cabernet) as a source of ACE inhibitory and antihypertensive peptides.

It is noteworthy that in the studies presented here, only Flavourzyme was used as an enzyme complex to optimize the extraction of bioactives from wine lees. This is certainly due to its action on the chemical structure of the cell wall of yeasts and bacteria present in wine lees [89]. The cell wall is rich in structural proteins [96], macromolecules susceptible to hydrolysis by proteases, such as Flavourzyme, which favors the weakening of its integrity, increasing its permeability and, as a consequence, increasing the yield of extraction of desired phenolic compounds [74].

Other enzymes (Glucanex^®^ and Mannaway^®^—β-1, 3 glucanase and β-1,4-mannanase activities, respectively) have also been used in the hydrolysis of wine lees with the aim of optimizing the extraction of anthocyanins from this matrix, resulting in an increase in the extraction yield of these compounds of around 85.6% when compared to traditional extraction methods [97]. Due to the exclusion criteria established in this research (articles published in scientific events), this work was not initially counted, but it brings important insights regarding the use of other enzyme sources applied to wine lees.

Although studies with enzymes have shown promise, there is a notable need for the development of new research aimed at evaluating how changes in process variables (enzyme, extraction temperature, enzyme–substrate contact time, enzyme–substrate ratio, medium pH) may impact the extraction of bioactive compounds from wine lees. In addition to these, wine lees can be produced at different stages of processing, presenting different compositions, especially regarding bioactive compounds. In this sense, studies with different lees can bring interesting results when subjected to enzymatic treatment.

#### 2.4.5. Membrane Application

The application of membrane processes for the separation, purification, and concentration of bioactive phenolic compounds from agri-food by-products has attracted increasing interest [49]. These processes present a highly effective alternative compared to conventional methodologies, due to their reduced operational and maintenance costs, mild operating conditions in terms of temperature and pressure, easy control, scalability, and the remarkable selectivity of the compounds. These characteristics mainly result in high-quality extracts [98].

Despite this, its applicability still faces limitations and disadvantages, such as the characteristics inherent to membranes, such as the size and geometry of the pores, and the size of the molecules that compose them [47]. In addition to these, the high susceptibility to fouling is one of the factors that impact the performance and application of the technology [99].

In extraction processes using membranes, emphasis should be given to pressure-controlled separation processes, such as microfiltration (MF), ultrafiltration (UF), and nanofiltration (NF), as such technologies have been extensively researched for the recovery of phenolic compounds from various agrifood residues, winery sludge [100], and wine lees [51]. These processes offer an effective and selective approach to obtain high-quality phenolic compounds; in terms of their application to wine lees, a total of four works (12.9%) were found that applied these technologies to extract a wide variety of compounds from wine lees.

In this context, Mejia et al. [46] applied ultrafiltration and nanofiltration membranes to pre-filtered wine lees from first fermentation (Sangiovese and Cabernet Sauvignon) with the aim of extracting phenolic compounds from this winery waste. Among the treatments analyzed, the use of ultrafiltration with cellulose acetate membranes (CA400-38) proved to be a promising technology for the extraction of bioactive compounds from wine lees, as it allowed the production of a permeate with high concentrations of total phenolic compounds (382.6 ± 25.1 mg GAE/L) and low concentrations of polysaccharides (92% retention) and mannoproteins (100% retention). Additionally, due to the diverse selectivity of the membranes used in the research, the authors suggest the combination of ultrafiltration and nanofiltration, in consecutive stages, to optimize the extraction of bioactive compounds from lees, producing concentrated solutions with high antioxidant activity.

The extraction of phenolic compounds, with their separation occurring mainly from carbohydrates, seems to be one of the main objectives when membranes are applied to wine lees. As in the previous work, Giacobbo; Bernardes; Pinho [48] applied ultrafiltration (ETNA01PP and ETNA10PP) and nanofiltration (NF270) membranes for this purpose in lees from malolactic fermentation (merlot). In this study, the authors also identified that ultrafiltration (UF) was able to separate polysaccharides from polyphenols, with the former remaining mainly in the reject stream (92.84%) and the polyphenols passing preferentially through the membrane. Additionally, it was observed that UF membranes (10 kDa) exhibited a higher permeation flux; on the other hand, nanofiltration membranes showed a high rejection coefficient of antioxidants, with 100% of anthocyanins and over 90% of total phenolic compounds being retained by the membrane. This behavior is important, especially when aiming for the integrated use of ultrafiltration and nanofiltration technologies to purify phenolic compounds present in wine lees. The extraction and application of these purified extracts produced with low energy consumption technologies, without additives, mild processing conditions, easy scalability, and efficient separation, may attract the interest of industries, such as pharmaceuticals, cosmetics, or food, for example.

Mir-Cerdà et al. [47], unlike other authors, initially prepared an aqueous extract (1:10, *w*/*w*) from the lees of malolactic fermentation wine (Albariño), followed by the extraction and purification of phenolic compounds from this extract using micro- and ultrafiltration (30 kDa and 5 kDa, respectively). The extract presented a varied composition, with phenolic acids being the main polyphenols detected in general, highlighting the presence of caffeic acid (200 µg/g), gallic acid (15 µg/g), and astilbin (40 µg/g) as the main compounds present. Additionally, the use of polyacrylonitrile UF membranes (30 kDa) did not alter the composition of the extract, allowing the removal of impurities, such as microparticles and macromolecules, producing an extract rich in phenolic compounds. The authors also suggest complementary purification and filtration processes (nanofiltration, chromatographic resins) for the permeate stream from the 30 kDa membrane to obtain more purified extracts of phenolic compounds from wine lees, adding value to this by-product.

On the other hand, Arboleda Meija et al. [49] aimed to develop a sustainable process for the recovery of phenolic compounds from red wine lees, obtained after alcoholic fermentation, through a combination of microwave extraction and membrane operations. Initially, the authors optimized the production of the extract by subjecting it to different microwave powers (90–350 W) for different periods (0.5–3 min), with the optimal condition being the use of powers in the order of 350 W for 2 min (933.9 ± 6.8 mg GAE/L) applied to the hydroalcoholic extract (75%). After this process, the authors microfiltered (polyvinylidene fluoride membrane, 0.15 μm) the extracts and processed them through three different polymeric membranes, ultrafiltration (Etna 01PP, composite fluorinated polymer, 1000 Da), and nanofiltration (NFT50—aromatic/aliphatic polyamide and Desal DK—Cross-linked aromatic polyamide, 150–300 Da). As a main result, the authors identified that the 150 Da polyamide membrane (NFT50) showed the highest retention in relation to total phenolic compounds (>55%) (feed: 857.00 mg GAE/L, retentate: 476.10 mg GAE/L), gallic acid (>70%) (feed: 16.50 mg/L, retentate: 11.80 mg/L), and catechin (>85%) (feed: 2.7 mg/L, retentate: 2.3 mg/L), thus maximizing the recovery of these compounds from the microfiltered extracts of wine lees.

In this sense, the authors bring a new perspective on the combined use of microwaves with filtration processes to optimize and obtain extracts with higher levels of phenolic compounds. Thus, it is observed that membrane extraction techniques appear to be interesting procedures when the aim is to extract bioactive compounds from wine lees, mainly due to their mild processing conditions (low temperatures and pH), often without the use of solvents, and they also have high efficiency in separating different target compounds. In terms of future trends, further research needs to focus primarily on optimizing membrane extraction conditions, assessing the effects of process variable modifications on the quantity and profile of compounds extracted by the technique. Additionally, the development of research with the application of complementary processes (such as ultrasound and enzymatic treatments) can also act to increase the efficiency of extraction and purification of phenolic compounds from wine lees, adding value to this by-product of the winemaking industry and allowing its application in different industrial segments.

#### 2.4.6. Supercritical and Pressurized Fluid Extraction

One of the techniques that has been used in recent years to optimize the extraction of bioactive compounds from a wide variety of agro-industrial residues [101,102,103] is supercritical fluid extraction (SFE) and pressurized fluid extraction (PFE), mainly due to their accessibility and the purity of the resulting products [104,105]. Additionally, these methods present several advantages, including greater extraction selectivity, higher purity of extracts, ease of solvent residue removal, and almost complete reuse of CO_2_ solvent through recirculation in the process line, which can be considered an economic advantage [106].

This method employs a solvent under conditions beyond its critical temperature and pressure [107], with carbon dioxide (CO_2_) being one of the main solvents used, mainly due to its low critical temperature (31.1 °C) and pressure (7.28 MPa). Additionally, its non-toxic and non-flammable nature makes CO_2_ environmentally friendly. Furthermore, its high volatility eliminates the need for complementary separation technologies [108].

Despite its routine use, supercritical CO_2_ has a high affinity for compounds with apolar characteristics, which may result in less effective removal of polar phenolic compounds. Therefore, the combined use of complementary technologies, such as pressurized fluid, seems to be an interesting alternative for isolating valuable ingredients [109].

Among the technologies employed for the extraction of bioactive compounds from wine lees, and considering the criteria established previously for this research, supercritical fluid extraction and pressurized fluid extraction were the least utilized techniques for extracting bioactive compounds from wine lees, as only 6.3% (*n* = 2) of the studies found applied the technique to extract any compound from wine lees.

Specifically, the study by Naziri et al. [50] aimed to optimize the extraction of squalene from wine lees using supercritical carbon dioxide (CO_2_) as the extraction solvent. By varying the pressure between 12 and 30 MPa under isothermal conditions (40 °C), the authors developed extracts from wine lees with squalene contents of around 16.9 g/kg, a value lower than that found using traditional methods (organic solvent acid extraction, 17.6 g/kg) and ultrasound-assisted extraction (20.4 g/kg of hexane extract) in the same study. High-performance liquid chromatography (HPLC) results did not detect squalene oxidation products in the extract developed using supercritical fluid, a significant advantage compared to other extraction methods due to the low pressures involved. As an alternative to optimize the extraction of these compounds from wine lees (mainly yeast cells), the authors suggest further research, including processes with high pressure (30 MPa) or ultrasound pretreatment to optimize the extraction system.

Tapia-Quirós et al. [110] applied a pressurized hydroalcoholic mixture (50:50, *v*/*v*) to red wine lees (Tempranillo) to evaluate the effects of high-pressure extraction techniques on total phenolic compound extraction. The authors applied pressures of 1500 psi for 5 min at a temperature of 100 °C and observed that the extract obtained had total phenolic compound contents ranging from 0.32 to 0.37 mg GAE/g fresh extract. It is important to highlight that, compared to treatments with ultrasound and microwave techniques, which were also applied in the study, the application of pressurized fluid provided higher extraction of the target compounds in the research.

Thus, it can be observed that the use of both supercritical fluids and pressurized solvents seems to be viable alternatives to optimize the extraction of bioactive compounds from wine lees. In this context, new studies need to be developed, mainly aiming at combining methods to ensure greater efficiency in the extraction of these compounds, as each agent employed has very specific selectivity, requiring a combined process to optimize the extraction systems of the active antioxidant constituents from residues.

#### 2.4.7. Average Extraction Yield of Bioactive Compounds

**Figure 9:**
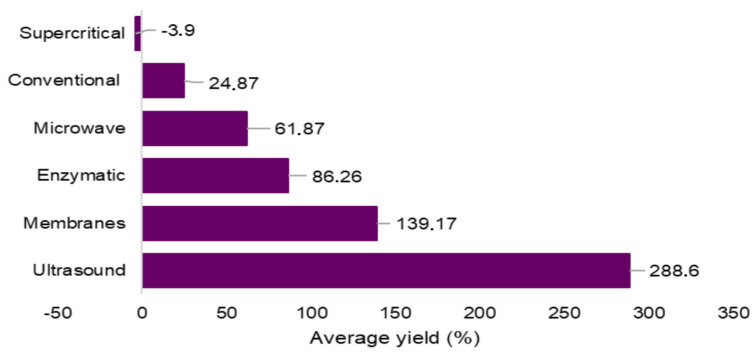
Average yields of bioactive extraction from various wine lees, determined through research or calculated as per Section 3.4.

Performing a general analysis of the extraction yield of bioactive from wine lees, it can be observed (Figure 9) that most of the complementary methods employed, whether involving ultrasound (288%), microwave (61.87%), membrane use (139.17%), or enzyme application (86.26%), provided much higher extraction yields than traditional methods (24.97%) for extracting bioactives from wine lees.

In this context, the application of ultrasound as a complementary method stands out, as in the studies found in this research, it was the method that provided the highest average yields of bioactive extraction from different wine lees, resulting in increases of up to 288% when compared to traditional methods.

This result can be directly attributed to the significant number of studies found in this research that used ultrasound as a complementary method in the extraction of bioactive compounds from wine lees. As mentioned earlier, this technique was the most employed in the extraction of bioactive compounds from this source. Furthermore, it is relevant to emphasize that ultrasound is recognized as an easy-to-operate technique, in addition to being accessible, simple, and efficient, which enables it to extract a wide variety of compounds under mild processing conditions [70]. These factors may have contributed to the prominence of this method in this research.

As shown in Figure 9, the supercritical fluid extraction method was the only one to present lower yields compared to traditional methods for the target molecule, registering a decrease of −3.8%. Nevertheless, it is important to highlight that this technique allowed the extraction of target compounds without inducing oxidative processes in the molecule, unlike conventional methods [50]. However, due to the scarcity of studies on this method, further research is needed.

Examples of such investigations include the studies conducted by Oliveira et al. [111] and Mihalcea et al. [112], in which the use of supercritical fluids on various residues from the wine industry resulted in extraction yields of bioactive compounds ranging between 1.99% and 5%. Special mention is given to the work of de Souza et al. [85], who, by applying supercritical fluids to grape seeds, obtained yields of vitamin E (α-tocopherol) ranging from 270.55% (Soxhlet extraction) to 348.35% (Bligh and Dyer method), demonstrating the potential of the method for extracting specific bioactive compounds from this residue.

#### 2.4.8. Insights into New Research

**Figure 10:**
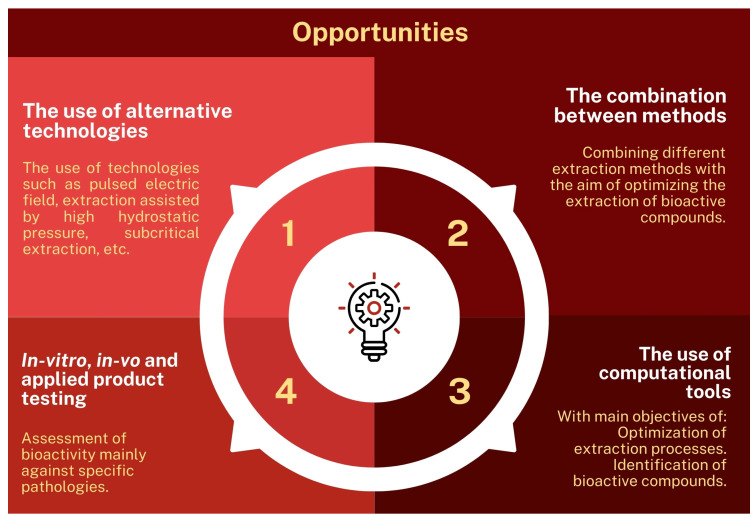
Key opportunities for future research applied to wine lees.

Figure 10 presents the main opportunities and insights generated throughout the reading of the texts in this systematic review, and, as can be observed, one of the main opportunities is linked to the use of alternative technologies aimed at optimizing the extraction of bioactive compounds and the consequent valorization of this residue from the wine industry. Technologies, such as the use of pulsed electric fields [113,114], the use of high hydrostatic pressures [115], subcritical extraction [116], and ohmic heating [117], have been demonstrated as viable alternatives for the extraction of bioactive compounds from a wide variety of agro-industrial residues [74,118].

Within the scope of alternative technologies, it is relevant to consider the feasibility of using alternative and green solvents for extracting bioactive from various wine lees. In this regard, the potential of using eutectic solvents and ionic liquids, either individually or in combination, as extracting solvents applied to wine lees deserves attention [119].

Moreover, the combination of methods seems to enhance the extraction efficiency of these compounds, especially when coupled with the use of computational tools capable of determining the optimal extraction conditions through computational simulations. These tools can even be used to optimize experimental design, employing more robust statistical tools, as seen in some studies on wine lees [36,37].

Furthermore, the use of computational tools can also contribute to in silico studies, aiding in the identification of compound profiles and elucidating the mechanisms of their bioactivity.

In this context, it is also suggested to conduct in vitro and in vivo tests to evaluate the bioactive potential of extracts from different wine lees. Moreover, such studies will allow for the assessment of the biosafety of these extracts, directly contributing to potential applications against a wide variety of pathologies.

## 3. Materials and Methods

For the development of this research, the recommendations of the Preferred Reporting Items for Systematic Reviews and Meta-Analyses (PRISMA) [20] were followed. The systematic review was structured with the assistance of the State of the Art through Systematic Review (StArt) software, version 3.0.4 Beta [120], and the collection of numerical data was performed directly from the texts. When graphically available, the Web Plot Digitizer version 4.5 [121] was used as a reverse engineering tool to obtain approximate values.

### 3.1. Database

For this research, the Science Citation Index Expanded (SCI-E)—Web of Science^®^ (https://www.webofscience.com, accessed between 12 and 15 November 2023) from Clarivate Analytics [122] was chosen as the electronic database for scientific journals, as it offers access to various databases and groups, including Elsevier, Springer, Wiley, Taylor & Francis, the Sage Group, and the Scientific Electronic Library Online (SciELO).

### 3.2. Search and Identification Strategies

The initial search strategy adopted the application of truncation techniques and Boolean operators, according to the characters and commands supported by the chosen database (Web of Science^®^). Initially, publications of scientific articles in events, technical books, monographs, dissertations, and theses were discarded through filters applied in the tool interface. At this point it was also possible to filter the research by year, and works published between 2013 and 2023 were selected.

Searches on the extraction of bioactive compounds from wine lees were conducted between 12 and 15 November 2023; as a strategy for refining the research, combined keywords with different Boolean operators and truncation techniques were used. Among these, the use of the Boolean operators AND and OR is notable, as in the search process they function to find records containing all or any of the keywords separated by the operators. Additionally, parentheses “( )” were used around the keywords as a mechanism to guide the application regarding the order of priority in the search process [123].

Additionally, to achieve better control over variations in plurals, truncation techniques, such as the use of asterisks () and quotation marks (“ ”) were employed. This allowed for an expanded search, taking into account prefix variations as well as the search for compound terms [123]. Thus, the following combined keywords were used in English: extraction AND “wine lees” AND (“bioactive compounds” OR bioactives OR “phenolic compounds” OR anthocyan).

### 3.3. Data Organization and Information Grouping

Data acquisition followed a three-step protocol: (i) verification of eligibility and exclusion criteria, (ii) screening, and (iii) the inclusion and selection of information. No bias risk determination was made. All (100%; *n* = 7) of the following questions comprised the eligibility and exclusion criteria: (1) Was the article published between 2013 and the current year 2023?; (2) Was any method of extracting bioactive compounds from wine lees applied?; (3) Was the extraction method well described?; (4) Was the method of identifying the compounds present well described?; (5) Were there any duplicated works?; (6) Is it an article published in the English language?; (7) Is it a review article?

In the first stage, articles that did not meet the eligibility criteria (i) were discarded, and the preliminary selection was made by reading the abstracts of the articles. Screening (ii) was conducted by reading the full texts. In the third stage (iii), the following information was extracted: (i) extraction method employed; (ii) production stage of the lees; (iii) varieties used; (iv) solvents employed; (v) extraction conditions; (vi) main identified compounds; (vii) antioxidant or bioactive activity. The information was grouped into topics and tables and analyzed according to the extraction techniques employed. It was also possible to categorize the information regarding the types of solvents employed, the types of lees, and the technological stage where these are produced.

### 3.4. Evaluation of the Average Yields of Extraction Processes

With the aim of understanding which method promotes higher extraction yields of different bioactive compounds from wine lees, a survey was conducted on the works selected in stage iii of the previous item regarding this extraction variable. When not explicitly described in the text, this was determined considering the values expressed in the text, with a conventional extraction method being used as the standard in each work.

The calculation to determine the extraction yield was performed according to the following Equation (1):

Extraction yield = [(concentration of the target compound by the alternative method)/(concentration of the target compound by the traditional method) × 100].(1)

It is important to note that when values were graphically expressed in the text, the Web Plot Digitizer version 4.5 [121] was used to extract the information through reverse engineering.

### 3.5. Bibliometric Characterization of the Data

Bibliometric analysis was conducted after transferring the bibliographic data from the Web of Science to the VOSviewer software (version 1.6.19). Each record contained comprehensive information, including authors, affiliations, countries/regions, title, abstract, keywords, year of publication, journal name, and references. References were detailed, including the name of the first author, year of publication, source type (journals, conference proceedings, books, etc.), volume number, and DOI. After collecting this information, an analysis was conducted to determine the profile of co-citations and to conduct bibliographic coupling of the previously selected research. This analysis resulted in the creation of a graph composed of three fields and a thematic map, highlighting the frequency and importance of keyword sets [124]. The extracted data were then analyzed using the “Bibliometrix” package in the R language. This analysis resulted in the creation of a graph with three fields and a thematic map, highlighting the frequency and importance of keyword sets [125,126].

## 4. Conclusions

This study aimed to outline the main methods used for extracting biologically active compounds from wine lees, a byproduct of the wine industry with a high concentration of bioactive compounds. A wide variety of methods were observed for extracting phenolic compounds from wine lees, with ultrasound standing out as the primary and most efficient method employed, particularly when compared to the traditional method (maceration).

The use of membranes, enzymatic methods, and supercritical fluid and pressurized methods have been applied as alternative techniques for extracting bioactive compounds from wine lees, but research is still scarce considering the technological and bioactive potential of lees. Additional research is needed to optimize the extraction of these compounds, including the combination of methods.

## Figures and Tables

**Table 1:** Identified studies with the methods and extraction conditions employed, as well as the main identified compounds.

Order	Extraction Method	Lees Type	Variety	Solvent Employed	Extraction Conditions	Compounds and/or Bioactive Activity Identified	Reference
1	Conventional	-	Whites (Sauvignon Blanc, Chardonnay, Pinot Gris (PG); reds (Pinot Noir (PN))	EtOH:H_2_O (50:50)	Maceration for 60 m	TPC (red wine lees—Pinot Noir—17.3 ± 0.4 and 40.9 ± 1.6 mg/g dry weigh; PN; rosé—9.8 ± 0.2 and 10.5 ± 0.5 mg/g FDM); white wine lees—(3.1 ± 0.2 and 10.3 ± 0.4 mg/g dry weigh).	[22]
2	Conventional	AF	White (Sauvignon Blanc); red and rosé (Tempranillo)	Methanol	Maceration for 12 h	ANT (rosé—1.147 ± 0.004; red—2.149 ± 0.059)	[23]
3	Conventional	-	White wine (Albariño); red wine (Tempranillo); rosé wine (Mencía)	Ultrapure water (green solvents)	Proportion 1:100 (lees–solvent Kg/L); temperature: 70 °C; 1 extraction cycle; pH 5.00	TPC (white wine (Albariño)—Ranging from 84.68 ± 0.47 to 172 ± 0.01; red wine (Tempranillo)—ranging from 341 ± 0.01 to 534 ± 0.20 mg GAE/Kg; rosé wine (Mencía) 48 ± 6 (mg EGA/Kg)	[24]
4	Conventional	-	-	EtOH:H_2_O (70:30 *v*/*v*) and 1.5 M hydrochloric acid, pH adjusted to 1.5	Solute–solvent 1:1.5 (m:v)	ANT (316.90 mg per 100 g of extract)	[25]
5	Conventional	AF	Malbec	EtOH:H_2_O acidified with HCl (75:25 *v*/*v*, pH 4)	Maceration for 60 min.	TPC (57 different types identified); ANT (a wide variety of anthocyanin derivatives, the pyranosanthocyanins)	[26]
6	Conventional	AF	Braca (Rabigato, Malvasia Fina, Viosinho); Tinta (Touriga Nacional, Touriga Franc, Tinta Roriz)	EtOH:H_2_O (50:50, *v*/*v*)	Maceration for 30 min.	TPC (32.26 mg EAG/g dry weight); TF (20 mg ECAT/g dry weight)	[27]
7	Ultrasound	AF	Merlot	Eutectic solvent (choline chloride (Ch), acidified with malic acid (Ma))	Water content in NADES: 35.4% Extraction time: 30.6 min Ultrasonic power: 341.5 W	ANT (6.55 mg malvidin-3-glucoside equivalents (mg/g))	[28]
8	Ultrasound	-	Merlot and Vranac	EtOH:H_2_O (50:50) acidified with 1.5% formic acid	Probe diameter (22 mm); amplitude (90%); extraction time (Vranac—1500 s; Merlot: 921.81 s to 1492.15 s)	TF (trans-resveratrol glucoside (300%), trans-resveratrol (45.75%), quercetin (43.83%), kaempferol (72.73%); ANT (petunidin-3-glucoside (64.95%), malvidin-3-glucoside (89.17%), malvidin-3-(6-O-acetyl)glucoside (49.74%), and malvidin-3-(6-O-p-coumaroyl) glycoside (34.93%))	[29]
9	Ultrasound	-	-	EtOH:H_2_O (80:20, *v*/*v*)	Extraction time (5 min); sonication (5 min)	TPC (WL (37.54 mg EAG/g extract); SL (26.66 mg EAG/g extract))	[30]
10	Ultrasound	MF	Tempranillo	EtOH:H_2_O (75:25, *v*/*v*)	Sonication (10 min, 700 Hz)	TPC (254 ± 24 mg EAG/g); FT (16 ± 1 to 146 ± 5 mg ECAT/g)	[31]
11	Ultrasound	AF	Cabernet Sauvignon; Cabernet Franc	Methanol, water, and formic acid (50:48.5:1.5 *v*/*v*)		ANT (19 anthocyanins and 9 pyranoanthocyanins were provisionally identified)	[32]
12	Ultrasound	MF	Cabernet Sauvignon 60%, Merlot 30%, Cabernet Franc 10%	EtOH:H_2_O (43.9%) EtOH:H_2_O (51.5%)	CFT (ultrasound frequency: 40 kHz; extraction time: 25 min and 36.3 min; temperature: 60 °C; ethanol: 43.9%) ANT (extraction time: 36.3 min; temperature: 59.9 °C; solid–solvent: 60:1; ethanol: 51.5%)	TPC (58.76 ± 0.38 mg/g)	[33]
13	Ultrasound	AF	Nocera Rosso, Magliocco Rosato, Magliocco Canino, Gaglioppo	EtOH:H_2_O (50:50, *v*/*v*) acidified (pH 2)	Frequency: 40 KHz/15 min at 30 °C	TPC (from 4.61 ± 0.09 to 49.56 ± 0.56 mg GAE/g); TF (from 1.54 ± 0.07 to 20.15 ± 0.32 mg ECAT/g)	[34]
14	Microwave Ultrasound Supercritical Fluid	-	Tempranillo	EtOH:H_2_O (50:50, *v*/*v*)	Ultrasound (30 min; 1:20 g/mL (sample–solvent)); microwave (5 min, 1:20 g/mL, (sample–solvent); temperature: 90 °C) Pressurized liquid (5 min, pressure: 1500 PSI, temperature: 100 °C)	TPC (US—0.27 to 30 mg GAE/g fresh extract; MO—0.31 to 33 mg GAE/g fresh extract; FP—0.32 to 0.37 mg GAE/g fresh extract)	[24]
15	Ultrasound	MF	Tempranillo	MeOH:H_2_O:formic acid (75:24.9:0.1; *v*/*v*/*v*)	Sonication 30 °C/30 min	TPC (42,330 ± 2963 µg GAE/g)	[35]
16	Ultrasound	AF and MF	Syrah and Cabernet Sauvignon	EtOH:H_2_O (50:50, *v*/*v*)	Amplitude of 53%, with cycle 0.30 s^−1^/ 10 min at 40 °C	ANT (148.03 ± 1.71 mg/100 g)	[36]
17	Microwave	-	Whites (Kidonitsa, Savvatiano, Chardonnay, Moschofilero); reds (Red Garnet 1, Red Garnet 2, Merlot, Cabernet, Agiorgitiko)	EtOH:H_2_O (50:50, *v*/*v*)	Power: 54 W/35 min; temperature: 85 °C	TPC (red wine—8.0 (±1.4) to 26.0 (±1.1) mg GAE/g of dry sediment); white wine—3.57 (±0.40) to 13.22 (±0.79) mg of ET/g of dry sediment.)	[37]
18	Microwave	AF	-	EtOH:H_2_O (60:40) (*v*/*v*) adjusted to pH 4 with formic acid	Power: 140 W/10 min	Flavonols (quercetin, quercetin 3-O-glucoside, myricetin, kaempferol and isorhamnetin, catechin, epicatechin, gallocatechin, procyanidin B2, and cinnamtannin); flavones (3,4,5-trimethoxyflavone); Anthocyanins (peonidin 3-O-glucoside, peonidin 3-(6-p—coumarylglucoside), malvidin 3-(6-p—coumarylglucoside))	[38]
19	Microwave	FA and MF	Tempranillo	EtOH:H_2_O (50:50, *v*/*v*)	Power: 300 W/90 s	TPC—(23.44 ± 0.11 to 42.04 ± 0.22 mg GAE/g); ANT (2.9 ± 0.2 to 6.2 ± 0.4 mg malvidin-equivalents/g)	[39]
20	Microwave	MF	Tempranillo; Mazuelo; Garnacha Cabernet	EtOH:H_2_O (60:40, *v*/*v*)—pH 4.0 with formic acid	Power 140 W/10 min	3,4,5-trimethoxyflavone and malvidin 3-(6-p-coumarylglucoside); two flavanol isomers, catechin and epicatechin; quercetin, myricetin, and conjugates, such as malvidin 3-galactoside; primary amino acid; valine	[40]
21	Microwave	-	White (Trebbiano); red and rosé (Nero di Troia)	EtOH:H_2_O (50:50, *v*/*v*)	Frequency 2450 MHz/10 min	TPC (white—64.60 (without Na_2_CO_3_ at 200 °C)—EtOH; rosé—85.48 ± 10.26 mg EAG/g dry weight (without Na_2_CO_3_ at 200 °C)—EtOH:H_2_O; red—33.62 (with Na_2_CO_3_ at 200 °C)—EtOH	[41]
22	Microwave	-	Tempranillo	EtOH:H_2_O (60:40, *v*/*v*)	Power of 300 W/90 s at 115 °C	TPC (266.0 ± 5.6 mg GAE/g); ANT (29.5 ± 2.3 mg of malv-3-O-gl/g extract)	[42]
23	Microwave	AF	Tempranillo; Mazuelo; Graciano Garnacha; Syrah; Cabernet Franc Merlot	EtOH:H_2_O (60:40, *v*/*v*) (*v*/*v*) adjusted to pH 4 with HCl	Power of 140 W/10 min	ANT (peonidin-3-glucoside, petunidin-3-glucoside, delphinidin-3-glucoside and delphinidin-3-rutinoside)	[43]
24	Enzymatic	AF	Cabernet	-	Enzyme–substrate ratio, 80 LAPU/g protein 25 °C/2 h, pH 4.0 and 250 rpm	TPC (33.52% of phenolic compounds (24.5 mg/g))	[44]
25	Enzymatic	-	Cabernet	-	Enzyme–substrate ratio, 80 LAPU/g protein 25 °C/2 h, pH 4.0 and 250 rpm	6 new peptides	[45]
26	Membranes	-	Sangiovese and Cabernet Sauvignon	-	UF (2 bar/25 ± 1 °C/0.55 L/min)	TPC mg GAE/L (UF—feed: 655.4 ± 13.6; permeate: 382.6 ± 25.1; retentate: 715.9 ± 44.5)	[46]
27	Membranes	MF	Albariño	Water (green solvent)	40 °C/30 min shaking	Caftaric acid (200 µg/g); transcoutaric acid, cis-coutaric acid, gallic acid, and astilbine with concentrations between 15 and 40 µg/g	[47]
28	Membranes	-	Merlot	-	Feeding speed 150 L/h	TPC (rejection coefficient—NF270—92.84%)	[48]
29	Membranes	AF	-	EtOH:H_2_O (75:25, *v*/*v*) with hydrochloric acid	Microwave (power: 350 W, time: 2 min); microfiltration (PVDF membrane, 0.15 μm) and nanofiltration (polyamide membrane, 150 Da) membranes	Microwave—TPC (933 GAE mg/L); membranes—TPC (4662.5 ± 224.8 GAE mg/L)	[49]
30	Supercritical Fluid	AF	Xynomavro	CO_2_	Temperature: 40 °C	Scalene (Total: 16.9 g/kg)	[50]

Where: “-”: information not disclosed; AF: alcoholic fermentation; MF: malolactic fermentation; EtOH: ethanol; H_2_O: water; ANT: anthocyanins; TPC: total phenolic compounds; TF: total flavonoids; GAE: gallic acid equivalent; ECAT; equivalent to catechin; WL: wet lees; SL: solid lees.

## Data Availability

Data presented are contained within the article.

## References

[1] Lavelli V., Zeppa G., Fiori L., Spigno G. (2016). Recovery of winemaking by-products for innovative food applications—A review. Ital. J. Food Sci..

[2] Bonamente E., Scrucca F., Asdrubali F., Cotana F., Presciutti A. (2015). The Water Footprint of the Wine Industry: Implementation of an Assessment Methodology and Application to a Case Study. Sustainability.

[3] de Andrade Bulos R.B., da Gama Paz F., Machado C.G., Tavares P.P.L.G., de Souza C.O., Umsza-Guez M.A. (2023). Scientific and Technological Research on the Use of Wine Lees. Food Prod. Process. Nutr..

[4] De Iseppi A., Marangon M., Vincenzi S., Lomolino G., Curioni A., Divol B. (2021). A Novel Approach for the Valorization of Wine Lees as a Source of Compounds Able to Modify Wine Properties. LWT.

[5] Bustamante M.A., Moral R., Paredes C., Pérez-Espinosa A., Moreno-Caselles J., Pérez-Murcia M.D. (2008). Agrochemical Characterisation of the Solid By-Products and Residues from the Winery and Distillery Industry. Waste Manag..

[6] Delteil D. (2002). Working with Lees: Key Elements to Wine Maturing. Aust. N. Z. Grapegrow. Winemak..

[7] Jara-Palacios M.J. (2019). Wine Lees as a Source of Antioxidant Compounds. Antioxidants.

[8] Cifuentes-Cabezas M., Pavani A., Vincent-Vela M.C., Mendoza-Roca J.A., Álvarez-Blanco S. (2023). Concentration of Phenolic Compounds from Olive Washing Wastewater by Forward Osmosis Using Table Olive Fermentation Brine as Draw Solution. Environ. Technol. Innov..

[9] Hapani U., Highland H., George L.-B. (2024). Phenolic Compounds: A Significant Threat to Agricultural Soils. Bioremediation of Emerging Contaminants from Soils.

[10] Cui W., Xu B., Chen F., Shen W., Wan F., Cheng A. (2024). Effects of Grape Peel Phenolics on Lipid Accumulation in Sodium Palmitate-Treated HepG2 Cells. J. Funct. Foods.

[11] Sinrod A.J.G., Shah I.M., Surek E., Barile D. (2023). Uncovering the Promising Role of Grape Pomace as a Modulator of the Gut Microbiome: An in-Depth Review. Heliyon.

[12] de Brito Alves J.L., Alves Brasil J.M., Maia L.A., Lima M.d.C., Sampaio K.B., de Souza E.L. (2023). Phenolic Compounds in Hypertension: Targeting Gut-Brain Interactions and Endothelial Dysfunction. J. Funct. Foods.

[13] Martín-Garcia A., Riu-Aumatell M., López-Tamames E. (2022). By-Product Revalorization: Cava Lees Can Improve the Fermentation Process and Change the Volatile Profile of Bread. Foods.

[14] Caponio G.R., Miolla R., Vacca M., Difonzo G., De Angelis M. (2024). Wine Lees as Functional Ingredient to Produce Biscuits Fortified with Polyphenols and Dietary Fibre. LWT.

[15] Hwang J.-Y., Shyu Y.-S., Hsu C.-K. (2009). Grape Wine Lees Improves the Rheological and Adds Antioxidant Properties to Ice Cream. LWT—Food Sci. Technol..

[16] Borges M.S., Biz A.P., Bertolo A.P., Bagatini L., Rigo E., Cavalheiro D. (2021). Enriched Cereal Bars with Wine Fermentation Biomass. J. Sci. Food Agric..

[17] Berk Z. (2009). Food Process Engineering and Technology.

[18] Jha A.K., Sit N. (2022). Extraction of Bioactive Compounds from Plant Materials Using Combination of Various Novel Methods: A Review. Trends Food Sci. Technol..

[19] Feitosa B.F., Decker B.L.A., Brito E.S.d., Rodrigues S., Mariutti L.R.B. (2023). Microencapsulation of Anthocyanins as Natural Dye Extracted from Fruits—A Systematic Review. Food Chem..

[20] Page M.J., McKenzie J.E., Bossuyt P.M., Boutron I., Hoffmann T.C., Mulrow C.D., Shamseer L., Tetzlaff J.M., Akl E.A., Brennan S.E. (2021). The PRISMA 2020 Statement: An Updated Guideline for Reporting Systematic Reviews. Syst. Rev..

[21] International Organisation of Vine and Wine World Wine Production Outlook 2023. https://www.oiv.int/sites/default/files/documents/OIV_World_Wine_Production_Outlook_2023_2.pdf.

[22] Zhijing Y., Shavandi A., Harrison R., Bekhit A.E.-D. (2018). Characterization of Phenolic Compounds in Wine Lees. Antioxidants.

[23] Sancho-Galán P., Amores-Arrocha A., Jiménez-Cantizano A., Palacios V. (2020). Physicochemical and Nutritional Characterization of Winemaking Lees: A New Food Ingredient. Agronomy.

[24] Tapia-Quirós P., Montenegro-Landívar M.F., Vecino X., Alvarino T., Cortina J.L., Saurina J., Granados M., Reig M. (2022). A Green Approach to Phenolic Compounds Recovery from Olive Mill and Winery Wastes. Sci. Total Environ..

[25] Aguiar G.P.S., Magro C.D., Carvalho G.O., Santos A.E., Lanza M., Oliveira J.V. (2021). Co-Precipitation of Anthocyanin in PHBV by the SEDS Technique. J. Food Sci. Technol..

[26] Fontana A., Schieber A. (2023). Preparative Fractionation of Phenolic Compounds and Isolation of an Enriched Flavonol Fraction from Winemaking Industry By-Products by High-Performance Counter-Current Chromatography. Plants.

[27] Costa R.D., Domínguez-Perles R., Abraão A., Gomes V., Gouvinhas I., Barros A.N. (2023). Exploring the Antioxidant Potential of Phenolic Compounds from Winery By-Products by Hydroethanolic Extraction. Molecules.

[28] Bosiljkov T., Dujmić F., Cvjetko Bubalo M., Hribar J., Vidrih R., Brnčić M., Zlatic E., Radojčić Redovniković I., Jokić S. (2017). Natural Deep Eutectic Solvents and Ultrasound-Assisted Extraction: Green Approaches for Extraction of Wine Lees Anthocyanins. Food Bioprod. Process..

[29] Dujmić F., Kovačević Ganić K., Ćurić D., Karlović S., Bosiljkov T., Ježek D., Vidrih R., Hribar J., Zlatić E., Prusina T. (2020). Non-Thermal Ultrasonic Extraction of Polyphenolic Compounds from Red Wine Lees. Foods.

[30] Duarte C.N., Taofiq O., Dias M.I., Heleno S.A., Santos-Buelga C., Barros L., Amaral J.S. (2023). Chemical Characterization and Bioactive Properties of Wine Lees and Diatomaceous Earth towards the Valorization of Underexploited Residues as Potential Cosmeceuticals. Cosmetics.

[31] Romero-Díez R., Rodríguez-Rojo S., Cocero M.J., Duarte C.M.M., Matias A.A., Bronze M.R. (2018). Phenolic Characterization of Aging Wine Lees: Correlation with Antioxidant Activities. Food Chem..

[32] Barcia M.T., Pertuzatti P.B., Rodrigues D., Gómez-Alonso S., Hermosín-Gutiérrez I., Godoy H.T. (2014). Occurrence of Low Molecular Weight Phenolics in Vitis Vinifera Red Grape Cultivars and Their Winemaking By-Products from São Paulo (Brazil). Food Res. Int..

[33] Tao Y., Wu D., Zhang Q.-A., Sun D.-W. (2014). Ultrasound-Assisted Extraction of Phenolics from Wine Lees: Modeling, Optimization and Stability of Extracts during Storage. Ultrason. Sonochem.

[34] De Luca M., Restuccia D., Spizzirri U.G., Crupi P., Ioele G., Gorelli B., Clodoveo M.L., Saponara S., Aiello F. (2023). Wine Lees as Source of Antioxidant Molecules: Green Extraction Procedure and Biological Activity. Antioxidants.

[35] Caro M., Sansone A., Amezaga J., Navarro V., Ferreri C., Tueros I. (2017). Wine Lees Modulate Lipid Metabolism and Induce Fatty Acid Remodelling in Zebrafish. Food Funct..

[36] Umsza-Guez M.A., Vázquez-Espinosa M., Chinchilla N., Aliaño-González M.J., Oliveira de Souza C., Ayena K., Fernández Barbero G., Palma M., Carrera C. (2023). Enhancing Anthocyanin Extraction from Wine Lees: A Comprehensive Ultrasound-Assisted Optimization Study. Antioxidants.

[37] Tagkouli D., Tsiaka T., Kritsi E., Soković M., Sinanoglou V.J., Lantzouraki D.Z., Zoumpoulakis P. (2022). Towards the Optimization of Microwave-Assisted Extraction and the Assessment of Chemical Profile, Antioxidant and Antimicrobial Activity of Wine Lees Extracts. Molecules.

[38] Delgado de la Torre M.P., Priego-Capote F., Luque de Castro M.D. (2015). Tentative Identification of Polar and Mid-polar Compounds in Extracts from Wine Lees by Liquid Chromatography–Tandem Mass Spectrometry in High-resolution Mode. J. Mass Spectrom..

[39] Romero-Díez R., Matos M., Rodrigues L., Bronze M.R., Rodríguez-Rojo S., Cocero M.J., Matias A.A. (2019). Microwave and Ultrasound Pre-Treatments to Enhance Anthocyanins Extraction from Different Wine Lees. Food Chem..

[40] Delgado de la Torre M.P., Priego-Capote F., Luque de Castro M.D. (2015). Characterization and Comparison of Wine Lees by Liquid Chromatography–Mass Spectrometry in High-Resolution Mode. J. Agric. Food Chem..

[41] Ciliberti M.G., Francavilla M., Albenzio M., Inghese C., Santillo A., Sevi A., Caroprese M. (2022). Green Extraction of Bioactive Compounds from Wine Lees and Their Bio-Responses on Immune Modulation Using in Vitro Sheep Model. J. Dairy. Sci..

[42] Matos M.S., Romero-Díez R., Álvarez A., Bronze M.R., Rodríguez-Rojo S., Mato R.B., Cocero M.J., Matias A.A. (2019). Polyphenol-Rich Extracts Obtained from Winemaking Waste Streams as Natural Ingredients with Cosmeceutical Potential. Antioxidants.

[43] Delgado de la Torre M.P., Ferreiro-Vera C., Priego-Capote F., Luque de Castro M.D. (2013). Anthocyanidins, Proanthocyanidins, and Anthocyanins Profiling in Wine Lees by Solid-Phase Extraction–Liquid Chromatography Coupled to Electrospray Ionization Tandem Mass Spectrometry with Data-Dependent Methods. J. Agric. Food Chem..

[44] López-Fernández-Sobrino R., Margalef M., Torres-Fuentes C., Ávila-Román J., Aragonès G., Muguerza B., Bravo F.I. (2021). Enzyme-Assisted Extraction to Obtain Phenolic-Enriched Wine Lees with Enhanced Bioactivity in Hypertensive Rats. Antioxidants.

[45] Bravo F.I., Mas-Capdevila A., López-Fernández-Sobrino R., Torres-Fuentes C., Mulero M., Alcaide-Hidalgo J.M., Muguerza B. (2022). Identification of Novel Antihypertensive Peptides from Wine Lees Hydrolysate. Food Chem..

[46] Mejia J.A.A., Ricci A., Figueiredo A.S., Versari A., Cassano A., de Pinho M.N., Parpinello G.P. (2022). Membrane-Based Operations for the Fractionation of Polyphenols and Polysaccharides From Winery Sludges. Food Bioproc. Tech..

[47] Mir-Cerdà A., Carretero I., Coves J.R., Pedrouso A., Castro-Barros C.M., Alvarino T., Cortina J.L., Saurina J., Granados M., Sentellas S. (2023). Recovery of Phenolic Compounds from Wine Lees Using Green Processing: Identifying Target Molecules and Assessing Membrane Ultrafiltration Performance. Sci. Total Environ..

[48] Giacobbo A., Bernardes A.M., de Pinho M.N. (2017). Sequential Pressure-Driven Membrane Operations to Recover and Fractionate Polyphenols and Polysaccharides from Second Racking Wine Lees. Sep. Purif. Technol..

[49] Arboleda Meija J.A., Parpinello G.P., Versari A., Conidi C., Cassano A. (2019). Microwave-Assisted Extraction and Membrane-Based Separation of Biophenols from Red Wine Lees. Food Bioprod. Process..

[50] Naziri E., Glisic S.B., Mantzouridou F.T., Tsimidou M.Z., Nedovic V., Bugarski B. (2016). Advantages of Supercritical Fluid Extraction for Recovery of Squalene from Wine Lees. J. Supercrit. Fluids.

[51] Giacobbo A., Dias B.B., Onorevoli B., Bernardes A.M., de Pinho M.N., Caramão E.B., Rodrigues E., Jacques R.A. (2019). Wine Lees from the 1st and 2nd Rackings: Valuable by-Products. J. Food Sci. Technol..

[52] Clodoveo M.L., Crupi P., Muraglia M., Corbo F. (2022). Ultrasound Assisted Extraction of Polyphenols from Ripe Carob Pods (*Ceratonia siliqua* L.): Combined Designs for Screening and Optimizing the Processing Parameters. Foods.

[53] Ivanović M., Albreht A., Krajnc P., Vovk I., Razboršek M.I. (2021). Sustainable Ultrasound-Assisted Extraction of Valuable Phenolics from Inflorescences of *Helichrysum arenarium* L. Using Natural Deep Eutectic Solvents. Ind. Crops Prod..

[54] Sittek L.-M., Schmidts T.M., Schlupp P. (2023). Potential Application of a Wine Extract in Skin Care: How to Benefit from the Antibacterial, Antioxidant and Elastase Inhibiting Properties. J. Cosmet. Dermatol. Sci. Appl..

[55] Emmulo E., Ceccantoni B., Bellincontro A., Mencarelli F. (2021). Use of Water and Ethanol Extracts from Wine Grape Seed Pomace to Prepare an Antioxidant Toothpaste. J. Sci. Food Agric..

[56] Carpes S.T., Pereira D., Moura C.d., dos Reis A.S., da Silva L.D., Oldoni T.L.C., Almeida J.F., Plata-Oviedo M.V.S. (2020). Lyophilized and Microencapsulated Extracts of Grape Pomace from Winemaking Industry to Prevent Lipid Oxidation in Chicken Pâté. Braz. J. Food Technol..

[57] Tapia-Blácido D.R., Garcia A.L., Beitum L.R., Zitei-Baptista L.F., Aguilar P.F. (2023). Use of Biobased Materials from Agro-Industrial Residues in Food Packaging. Advanced Applications of Biobased Materials.

[58] Queiroz V.A.V., Alves M.P., de Oliveira K.G., Rocha M.C., Conceição R.R.P.d., Miguel R.d.A. (2014). Substituição de Metanol Por Água Na Extração de Antocianinas Totais de Glumas de Sorgo Para Uso Como Corante Alimentício. Boletim de Pesquisa e Desenvolvimento—Embrapa Milho e Sorgo.

[59] Hansen B.B., Spittle S., Chen B., Poe D., Zhang Y., Klein J.M., Horton A., Adhikari L., Zelovich T., Doherty B.W. (2021). Deep Eutectic Solvents: A Review of Fundamentals and Applications. Chem. Rev..

[60] He Q., Tang G., Hu Y., Liu H., Tang H., Zhou Y., Deng X., Peng D., Qian Y., Guo W. (2024). Green and Highly Effective Extraction of Bioactive Flavonoids from Fructus Aurantii Employing Deep Eutectic Solvents-Based Ultrasonic-Assisted Extraction Protocol. Ultrason. Sonochem..

[61] Abbott A.P., Boothby D., Capper G., Davies D.L., Rasheed R.K. (2004). Deep Eutectic Solvents Formed between Choline Chloride and Carboxylic Acids: Versatile Alternatives to Ionic Liquids. J. Am. Chem. Soc..

[62] Cvjetko Bubalo M., Ćurko N., Tomašević M., Kovačević Ganić K., Radojčić Redovniković I. (2016). Green Extraction of Grape Skin Phenolics by Using Deep Eutectic Solvents. Food Chem..

[63] Azwanida N.N. (2015). A Review on the Extraction Methods Use in Medicinal Plants, Principle, Strength and Limitation. Med. Aromat. Plants.

[64] Kamil Hussain M., Saquib M., Faheem Khan M. (2019). Techniques for Extraction, Isolation, and Standardization of Bio-Active Compounds from Medicinal Plants. Natural Bio-Active Compounds.

[65] Luque de Castro M.D., García-Ayuso L.E. (1998). Soxhlet Extraction of Solid Materials: An Outdated Technique with a Promising Innovative Future. Anal. Chim. Acta.

[66] Srivastava N., Singh A., Kumari P., Nishad J.H., Gautam V.S., Yadav M., Bharti R., Kumar D., Kharwar R.N. (2021). Advances in Extraction Technologies: Isolation and Purification of Bioactive Compounds from Biological Materials. Natural Bioactive Compounds.

[67] Blanco-Vega D., López-Bellido F.J., Alía-Robledo J.M., Hermosín-Gutiérrez I. (2011). HPLC–DAD–ESI-MS/MS Characterization of Pyranoanthocyanins Pigments Formed in Model Wine. J. Agric. Food Chem..

[68] Picó Y. (2013). Ultrasound-Assisted Extraction for Food and Environmental Samples. TrAC Trends Anal. Chem..

[69] González de Peredo A.V., Vázquez-Espinosa M., Piñeiro Z., Espada-Bellido E., Ferreiro-González M., Barbero G.F., Palma M. (2021). Development of a Rapid and Accurate UHPLC-PDA-FL Method for the Quantification of Phenolic Compounds in Grapes. Food Chem..

[70] Wang L., Weller C.L. (2006). Recent Advances in Extraction of Nutraceuticals from Plants. Trends Food Sci. Technol..

[71] Horžić D., Jambrak A.R., Belščak-Cvitanović A., Komes D., Lelas V. (2012). Comparison of Conventional and Ultrasound Assisted Extraction Techniques of Yellow Tea and Bioactive Composition of Obtained Extracts. Food Bioproc. Tech..

[72] Vázquez-Espinosa M., González de Peredo A.V., Ferreiro-González M., Carrera C., Palma M., Barbero G.F., Espada-Bellido E. (2019). Assessment of Ultrasound Assisted Extraction as an Alternative Method for the Extraction of Anthocyanins and Total Phenolic Compounds from Maqui Berries (*Aristotelia chilensis* (Mol.) Stuntz). Agronomy.

[73] Rockenbach I.I., Gonzaga L.V., Rizelio V.M., Gonçalves A.E.d.S.S., Genovese M.I., Fett R. (2011). Phenolic Compounds and Antioxidant Activity of Seed and Skin Extracts of Red Grape (*Vitis vinifera* and *Vitis labrusca*) Pomace from Brazilian Winemaking. Food Res. Int..

[74] Boateng I.D., Clark K. (2024). Trends in Extracting Agro-Byproducts’ Phenolics Using Non-Thermal Technologies and Their Combinative Effect: Mechanisms, Potentials, Drawbacks, and Safety Evaluation. Food Chem..

[75] José Aliaño González M., Carrera C., Barbero G.F., Palma M. (2022). A Comparison Study between Ultrasound–Assisted and Enzyme–Assisted Extraction of Anthocyanins from Blackcurrant (*Ribes nigrum* L.). Food Chem. X.

[76] Luque García J.L., de Castro M.D.L. (2002). Acceleration and Automation of Solid Sample Treatment.

[77] Pérez-Serradilla J.A., Luque de Castro M.D. (2011). Microwave-Assisted Extraction of Phenolic Compounds from Wine Lees and Spray-Drying of the Extract. Food Chem..

[78] Filly A., Fernandez X., Minuti M., Visinoni F., Cravotto G., Chemat F. (2014). Solvent-Free Microwave Extraction of Essential Oil from Aromatic Herbs: From Laboratory to Pilot and Industrial Scale. Food Chem..

[79] Tsiaka T., Sinanoglou V.J., Zoumpoulakis P. (2017). Extracting Bioactive Compounds from Natural Sources Using Green High-Energy Approaches: Trends and Opportunities in Lab- and Large-Scale Applications. Ingredients Extraction by Physicochemical Methods in Food.

[80] Ganzler K., Salgó A., Valkó K. (1986). Microwave Extraction. J. Chromatogr. A.

[81] Lin Y.-S., Chen H.-J., Huang J.-P., Lee P.-C., Tsai C.-R., Hsu T.-F., Huang W.-Y. (2017). Kinetics of Tyrosinase Inhibitory Activity Using *Vitis vinifera* Leaf Extracts. BioMed Res. Int..

[82] Álvarez A., Poejo J., Matias A.A., Duarte C.M.M., Cocero M.J., Mato R.B. (2017). Microwave Pretreatment to Improve Extraction Efficiency and Polyphenol Extract Richness from Grape Pomace. Effect on Antioxidant Bioactivity. Food Bioprod. Process..

[83] Ren B., Chen C., Li C., Fu X., You L., Liu R.H. (2017). Optimization of Microwave-Assisted Extraction of Sargassum Thunbergii Polysaccharides and Its Antioxidant and Hypoglycemic Activities. Carbohydr. Polym..

[84] IOVW Global Economic Vitiviniculture Data. https://www.oiv.int/.

[85] Cano-Lamadrid M., Artés-Hernández F. (2021). By-Products Revalorization with Non-Thermal Treatments to Enhance Phytochemical Compounds of Fruit and Vegetables Derived Products: A Review. Foods.

[86] PALOMERO F., MORATA A., BENITO S., GONZALEZ M., SUAREZLEPE J. (2007). Conventional and Enzyme-Assisted Autolysis during Ageing over Lees in Red Wines: Influence on the Release of Polysaccharides from Yeast Cell Walls and on Wine Monomeric Anthocyanin Content. Food Chem..

[87] Shahidi F., Yeo J. (2016). Insoluble-Bound Phenolics in Food. Molecules.

[88] Panja P. (2018). Green Extraction Methods of Food Polyphenols from Vegetable Materials. Curr. Opin. Food Sci..

[89] Fărcaș A.C., Socaci S.A., Nemeș S.A., Salanță L.C., Chiș M.S., Pop C.R., Borșa A., Diaconeasa Z., Vodnar D.C. (2022). Cereal Waste Valorization through Conventional and Current Extraction Techniques—An Up-to-Date Overview. Foods.

[90] Ozdal T., Capanoglu E., Altay F. (2013). A Review on Protein–Phenolic Interactions and Associated Changes. Food Res. Int..

[91] Radenkovs V., Juhnevica-Radenkova K., Górnaś P., Seglina D. (2018). Non-Waste Technology through the Enzymatic Hydrolysis of Agro-Industrial by-Products. Trends Food Sci. Technol..

[92] Ozón B., Cotabarren J., Valicenti T., Graciela Parisi M., David Obregón W. (2022). Chia Expeller: A Promising Source of Antioxidant, Antihypertensive and Antithrombotic Peptides Produced by Enzymatic Hydrolysis with Alcalase and Flavourzyme. Food Chem..

[93] Zhang X., Yang J., Suo H., Tan J., Zhang Y., Song J. (2023). Identification and Molecular Mechanism of Action of Antibacterial Peptides from Flavourzyme-Hydrolyzed Yak Casein against Staphylococcus Aureus. J. Dairy. Sci..

[94] Meini M.-R., Cabezudo I., Boschetti C.E., Romanini D. (2019). Recovery of Phenolic Antioxidants from Syrah Grape Pomace through the Optimization of an Enzymatic Extraction Process. Food Chem..

[95] Gligor O., Mocan A., Moldovan C., Locatelli M., Crișan G., Ferreira I.C.F.R. (2019). Enzyme-Assisted Extractions of Polyphenols—A Comprehensive Review. Trends Food Sci. Technol..

[96] Ferracini-Santos L., Sato H.H. (2009). Isolamento de Polímeros Da Parede Celular de Saccharomyces Cerevisiae e Avaliação Da Atividade Antioxidante Da Manana-Proteína Isolada. Quim. Nova.

[97] Romero Díez R., Rodrigues L., Rodríguez Rojo S. Wine Lees Valorization: Pretreatment Effect on Anthocyanin Extraction Kinetics from Different Wine Lees. Proceedings of the 13th International Conference on Renewable Resources and Biorefineries.

[98] Castro-Muñoz R., Fíla V., Barragán-Huerta B.E., Yáñez-Fernández J., Piña-Rosas J.A., Arboleda-Mejía J. (2018). Processing of Xoconostle Fruit (*Opuntia joconostle* ) Juice for Improving Its Commercialization Using Membrane Filtration. J. Food Process Preserv..

[99] Garcia-Castello E.M., Conidi C., Cassano A. (2024). A Membrane-Assisted Green Strategy for Purifying Bioactive Compounds from Extracted White Wine Lees. Sep. Purif. Technol..

[100] Galanakis C.M., Markouli E., Gekas V. (2013). Recovery and Fractionation of Different Phenolic Classes from Winery Sludge Using Ultrafiltration. Sep. Purif. Technol..

[101] Cerón-Martínez L.J., Hurtado-Benavides A.M., Ayala-Aponte A., Serna-Cock L., Tirado D.F. (2021). A Pilot-Scale Supercritical Carbon Dioxide Extraction to Valorize Colombian Mango Seed Kernel. Molecules.

[102] de Souza R.d.C., Machado B.A.S., Barreto G.d.A., Leal I.L., dos Anjos J.P., Umsza-Guez M.A. (2020). Effect of Experimental Parameters on the Extraction of Grape Seed Oil Obtained by Low Pressure and Supercritical Fluid Extraction. Molecules.

[103] Sarkar R., Kundu A., Dutta A., Mandal A., Saha S. (2022). Citrus Peel as a Source for Waste Valorization and Its Greener Processing.

[104] Machado B.A.S., Pereira C.G., Nunes S.B., Padilha F.F., Umsza-Guez M.A. (2013). Supercritical Fluid Extraction Using CO_2_: Main Applications and Future Perspectives. Sep. Sci. Technol..

[105] Ray A., Dubey K.K., Marathe S.J., Singhal R. (2023). Supercritical Fluid Extraction of Bioactives from Fruit Waste and Its Therapeutic Potential. Food Biosci..

[106] Pereira G.S.L., Magalhães R.d.S., Fraga S., de Souza P.T., de Lima J.P., Meirelles A.J.d.A., Sampaio K.A. (2023). Extraction of Bioactive Compounds from *Butia capitata* Fruits Using Supercritical Carbon Dioxide and Pressurized Fluids. J. Supercrit. Fluids.

[107] Zulkafli Z.D., Wang H., Miyashita F., Utsumi N., Tamura K. (2014). Cosolvent-Modified Supercritical Carbon Dioxide Extraction of Phenolic Compounds from Bamboo Leaves (*Sasa palmata*). J. Supercrit. Fluids.

[108] Berna A., Cháfer A., Montón J.B., Subirats S. (2001). High-Pressure Solubility Data of System Ethanol (1)+catechin (2)+CO_2_ (3). J. Supercrit. Fluids.

[109] Kitrytė V., Bagdonaitė D., Rimantas Venskutonis P. (2018). Biorefining of Industrial Hemp (*Cannabis sativa* L.) Threshing Residues into Cannabinoid and Antioxidant Fractions by Supercritical Carbon Dioxide, Pressurized Liquid and Enzyme-Assisted Extractions. Food Chem..

[110] Tapia-Quirós P., Montenegro-Landívar M.F., Reig M., Vecino X., Alvarino T., Cortina J.L., Saurina J., Granados M. (2020). Olive Mill and Winery Wastes as Viable Sources of Bioactive Compounds: A Study on Polyphenols Recovery. Antioxidants.

[111] Oliveira D.A., Salvador A.A., Smânia A., Smânia E.F.A., Maraschin M., Ferreira S.R.S. (2013). Antimicrobial Activity and Composition Profile of Grape (*Vitis vinifera*) Pomace Extracts Obtained by Supercritical Fluids. J. Biotechnol..

[112] Mihalcea L., Coman G., Constantin O.E., Grigore-Gurgu L., Dănilă G.-M., Cucolea E.I., Turturică M., Nicoleta S. (2023). Conjugates-Based Design for Microencapsulation of CO_2_ Supercritical Extract from Red Grape by-Products to Provide Functional Ingredients. LWT.

[113] Comuzzo P., Marconi M., Zanella G., Querzè M. (2018). Pulsed Electric Field Processing of White Grapes (Cv. Garganega): Effects on Wine Composition and Volatile Compounds. Food Chem..

[114] Naliyadhara N., Kumar A., Girisa S., Daimary U.D., Hegde M., Kunnumakkara A.B. (2022). Pulsed Electric Field (PEF): Avant-Garde Extraction Escalation Technology in Food Industry. Trends Food Sci. Technol..

[115] Corrales M., Toepfl S., Butz P., Knorr D., Tauscher B. (2008). Extraction of Anthocyanins from Grape By-Products Assisted by Ultrasonics, High Hydrostatic Pressure or Pulsed Electric Fields: A Comparison. Innov. Food Sci. Emerg. Technol..

[116] Mok H.-W., Ko M.-J., Choi H.-J., Chung M.-S. (2022). Extraction of Chlorogenic Acids from Hibiscus (*Hibiscus syriacus* L.) by Subcritical-Water. J. Ind. Eng. Chem..

[117] Ferreira-Santos P., Nobre C., Rodrigues R.M., Genisheva Z., Botelho C., Teixeira J.A. (2024). Extraction of Phenolic Compounds from Grape Pomace Using Ohmic Heating: Chemical Composition, Bioactivity and Bioaccessibility. Food Chem..

[118] Chakka A.K., Babu A.S. (2022). Bioactive Compounds of Winery By-Products: Extraction Techniques and Their Potential Health Benefits. Appl. Food Res..

[119] Choi Y.H., Verpoorte R. (2019). Green Solvents for the Extraction of Bioactive Compounds from Natural Products Using Ionic Liquids and Deep Eutectic Solvents. Curr. Opin. Food Sci..

[120] Hernandes E., Zamboni A., Fabbri S., THOMMAZO A. (2012). Di Using GQM and TAM to Evaluate StArt—A Tool That Supports Systematic Review. CLEI Electron. J..

[121] Rohatgi A. WebPlotDigitizer 4.6 Retrieved 2022. https://automeris.io/WebPlotDigitizer.

[122] Wos Web of Science, Clarivate Analytics. https://www.webofscience.com.

[123] (2020). Clarivate Analytics Ferramentas WoS: Treinamento e Dicas. https://clarivate.com/webofsciencegroup/wp-content/uploads/sites/2/2020/06/CAPES_Ferramentas-WoS-treinamento-Dicas-tutorial_2020-002.pdf.

[124] Aria M., Cuccurullo C. (2017). Bibliometrix: An R-Tool for Comprehensive Science Mapping Analysis. J. Informetr..

[125] Ferreira V.C., Ampese L.C., Sganzerla W.G., Colpini L.M.S., Forster-Carneiro T. (2023). An Updated Review of Recent Applications and Future Perspectives on the Sustainable Valorization of Pitaya (*Hylocereus* spp.) by-Products. Sustain. Chem. Pharm..

[126] van Eck N.J., Waltman L. (2010). Software Survey: VOSviewer, a Computer Program for Bibliometric Mapping. Scientometrics.

